# Efficacy of Chinese traditional patent medicines for heart failure with preserved ejection fraction: a Bayesian network meta-analysis of 64 randomized controlled trials

**DOI:** 10.3389/fcvm.2023.1255940

**Published:** 2023-11-20

**Authors:** Hongxin Guo, Mingjun Zhu, Rui Yu, Xingyuan Li, Qifei Zhao

**Affiliations:** ^1^ First Clinical Medical College, Henan University of Traditional Chinese Medicine, Zhengzhou, China; ^2^ Department of Cardiovascular Diseases, The First Affiliated Hospital of Henan University of Traditional Chinese Medicine, Zhengzhou, China

**Keywords:** traditional Chinese medicine, Chinese traditional patent medicine, heart failure with preserved ejection fraction, randomized controlled trial, network meta-analysis, Bayesian model

## Abstract

**Background:**

Heart failure with preserved ejection fraction (HFpEF) is associated with substantial morbidity and mortality, and modern medicine offers less effective treatment for HFpEF. Much evidence shows that Chinese traditional patent medicines (CTPMs) have good efficacy for HFpEF, but the advantages and disadvantages of different CTPMs for HFpEF are still unclear. This study used network meta-analysis (NMA) to compare clinical efficacies of different CTPMs for HFpEF.

**Methods:**

Randomized controlled trials (RCTs) of CTPMs for treating HFpEF were searched in seven Chinese and English databases from inception to September 2023: China National Knowledge Infrastructure (CNKI), Wanfang, VIP, China Biology Medicine, PubMed, Cochrane Library, and Embase. Two researchers independently screened the literature, extracted data, and evaluated the quality of the included studies. The GeMTC package in R (version 4.1.2) was used to perform Bayesian NMA.

**Results:**

A total of 64 RCTs were included, involving six CTPMs and 6,238 patients. The six CTPMs were Qili Qiangxin capsule (QLQXC), Qishen Yiqi dropping pill (QSYQDP), Yixinshu capsule (YXSC), Yangxinshi tablet (YXST), Shexiang Baoxin Pill (SXBXP), and Tongxinluo capsule (TXLC). Conventional Western medicine (CWM) treatment was given to the control group, and CWM treatment combined with CTPM treatment was given to the experimental group. The results indicated that CPTMs + CWM were all superior to CWM alone; SXBXP + CWM had the best efficacies in improving the New York Heart Association cardiac functional classification efficiency; TXLC + CWM was best at improving the ratio of early diastolic mitral inflow velocity to late diastolic mitral inflow velocity (E/A); QSYQDP + CWM was best at reducing N-terminal pro-B type natriuretic peptide (NT-proBNP); and QSYQDP + CWM was best at improving the 6-min walking test. In terms of safety, there was no signiﬁcant difference between CTPMs + CWM and CWM.

**Conclusion:**

Compared with CWM alone, CTPMs + CWM combinations have certain advantages and good safety in the treatment of HFpEF. QSYQDP + CWM and SXBXP + CWM may be the potential optimal integrative medicine-based treatments for HFpEF. Given the limitations of this study, further high-quality, multicenter, large sample, randomized, and double-blind studies are needed to confirm the current results.

**Systematic Review Registration:**

identifier, CRD42022303938.

## Introduction

Heart failure (HF) is a group of complex clinical syndromes resulting from ventricular systolic and/or diastolic dysfunction caused by abnormal changes in cardiac structure or function for various reasons. HF can be categorized into heart failure with preserved ejection fraction [HFpEF, left ventricular ejection fraction (LVEF) ≥ 50%], heart failure with mid-range ejection fraction (HFmrEF, LVEF 40%–49%), and heart failure with reduced ejection fraction (HFrEF, LVEF < 40%) (1). With the increase in population aging and continuous increase in the number of individuals with chronic diseases such as obesity, diabetes mellitus, and hypertension, HFpEF has become the most important component of HF (2). Epidemiological surveys have shown that more than half of HF patients have HFpEF, with this proportion increasing yearly (3, 4). HFpEF has HF-related symptoms and signs, which are mainly characterized by ventricular diastolic dysfunction, decreased compliance, and increased stiffness. Although HFpEF patients have normal LVEF, their rehospitalization and mortality rates are comparable to those of HFrEF patients, placing serious economic and psychological burdens on patients and their families (5, 6).

HFpEF is mainly treated with comprehensive methods that target symptoms, cardiovascular diseases, comorbidities, and risk factors (7). The current guideline-recommended pharmacological treatments mainly include diuretics, sodium-glucose cotransporter−2 inhibitors, angiotensin receptor neprilysin inhibitors, mineralocorticoid receptor antagonists and angiotensin II receptor blockers. Nevertheless, only diuretics are recommended as class 1 for symptomatic relief with risks of electrolyte disturbances and renal impairment, and less treatment measure is available that reduces the hospitalization and mortality rates associated with HFpEF (8). In China, traditional Chinese medicine (TCM) has been used to treat cardiovascular diseases for more than 2,000 years. Several studies have systematically evaluated the efficacy and safety of different Chinese traditional patent medicines (CTPMs) in the treatment of HFpEF, showing that conventional Western medicine (CWM) combined with CTPMs can significantly alleviate symptoms and improve efficacyin HFpEF (9–11). Although many CTPMs are available on the market, there are still no clinical studies directly comparing the efficacies of different CTPMs. Therefore, it is difficult for clinicians to choose the best treatment, a situation that is not conducive to the promotion and application of CTPMs in the treatment of HFpEF. Unlike conventional meta-analysis, network meta-analysis (NMA) considers both direct evidence and indirect evidence to evaluate the therapeutic effects of various interventions and rank them according to efficacy, providing a reference and a basis for a clinical intervention, such as CTPMs. Given this background, this study used NMA to compare and rank the differences in the therapeutic effects of various CTPMs for treating HFPEF with regard to different outcome indicators to provide medical evidence for the development of clinical treatment plans.

## Information and methods

The Preferred Reporting Items for Systematic Reviews and Meta-Analyses (PRISMA) criteria (12) were followed while conducting this NMA (Supplementary Material S1), which was registered in the International Prospective Register of Systematic Reviews (number: CRD42022303938). The abbreviations used in this manuscript are listed in Tables 1, 2.

**Table 1:** Abbreviations of academic terms used in this manuscript.

Abbreviations	Full name
CI	Confidence interval
CNKI	China national knowledge infrastructure
HF	Heart failure
HFmrEF	Heart failure with mid-range ejection fraction
HFpEF	Heart failure with preserved ejection fraction
HFrEF	Heart failure with reduced ejection fraction
LVEF	Left ventricular ejection fraction
NMA	Network meta-analysis
NMPA	National medical products administration of China,
NYHA	New York Heart Association
OR	Odds ratio
PSRF	Potential scale reduction factor
RCTs	Randomized controlled trials
SUCRA	Surface under the cumulative ranking curve
TCM	Traditional Chinese medicine
WMD	Weighted mean difference

**Table 2:** Abbreviations of outcomes and interventions used in this manuscript.

Category	Abbreviations	Full name
Outcomes	NYHA efficiency	New York Heart Association cardiac functional classification efficiency
	E/A	The ratio of early diastolic mitral inflow velocity to late diastolic mitral inflow velocity
	NT-proBNP	N-terminal pro-B type natriuretic peptide
	6MWT	6-Minute walking test
	ADRs	adverse drug reactions
Interventions	CTPMs	Chinese traditional patent medicines
	CWM	Conventional Western medicine
	QLQXC	Qili Qiangxin capsule
	QSYQDP	Qishen Yiqi dropping pill
	SXBXP	Shexiang Baoxin Pill
	YXSC	Yixinshu capsule
	YXST	Yangxinshi tablet
	TXLC	Tongxinluo capsule

### Inclusion and exclusion criteria

#### Study type

Only RCTs written in Chinese and English were included in this NMA.

#### Participants

Patients in the stable or acute phase who met the diagnostic criteria for HFpEF were included without considering disease duration, sex, age, or region. The diagnostic criteria for HFpEF referred to *2021 ESC Guidelines for the diagnosis and treatment of acute and chronic heart failure* (1) or *Guidelines for diagnosis and treatment of heart failure in China 2018* (13).

#### Interventions and comparisons

Patients in the control group were treated with CWM or CWM plus placebo, and each CWM treatment followed the relevant guidelines for each medication. Basic drug therapy for HFpEF includes diuretics, mineralocorticoid receptor antagonist, angiotensin receptor blocker, angiotensin receptor nprilysin inhibitor, or sodium glucose cotransporter−2 inhibitor. Patients in the experimental group were treated with a CTPM combined with the treatment received by the control group. The CTPMs used were approved by the National Medical Products Administration of China (NMPA), with no restrictions regarding drug indications but with sufficient research data (≥5 RCTs for a single CTPM).

#### Outcomes

The first outcome was New York Heart Association cardiac functional classification efficiency (NYHA efficiency). The second outcome was the ratio of early diastolic mitral inflow velocity to late diastolic mitral inflow velocity (E/A). The third outcome was the N-terminal pro-B type natriuretic peptide (NT-proBNP). The fourth outcome was the 6-min walking test (6MWT). The fifth outcome was adverse drug reactions (ADRs). Improvement of the disease after drug treatment was assessed using NYHA efficiency according to guiding principles for clinical research of new Chinese medicines: improvement in NYHA cardiac function by ≥2 classes with significantly alleviated symptoms or signs was considered marked effectiveness; improvement in NYHA cardiac function by 1 class with alleviated symptoms or signs was considered effective; and no improvement in NYHA cardiac function with symptoms and signs unchanged or even worse than before treatment was considered ineffective. Overall effective rate = (markedly effectiveness + effective)/total number of cases × 100% (14).

#### Exclusion criteria

The following studies were excluded:

• RCTs involving patients with malignant arrhythmia, malignant tumors, severe liver or kidney dysfunction, or severe infections;
• RCTs involving non-CTPM preparations (such as decoctions, powders, and granules);
• RCTs in which the CTPMs used had not been approved by the NMPA of China;
• RCTs with obvious missing or erroneous research data;
• Duplicate RCTs with less comprehensive data; and

#### Search strategy

On the basis of relevant recommendations in Cochrane Handbook for Systematic Reviews of Interventions (15) and the inclusion and exclusion criteria, two researchers (GHX and LXY) independently searched China National Knowledge Infrastructure (CNKI), Wanfang Database, VIP Database, China Biology Medicine disc, PubMed, Cochrane Library, and Embase for relevant literature on CTPMs for treating HFpEF from inception until September 2023. The search terms included “Chinese Traditional Patent Medicine”, “heart failure with preserved ejection fraction”, “diastolic heart failure” and “clinical trial”, as well as their synonyms. Taking PubMed as an example, a detailed search strategy is shown in Box 1.

Box 1 **PubMed search strategy**

**#1** “heart failure, diastolic"[MeSH Terms]
**#2** “heart failure"[Title/Abstract] OR “cardiac failure"[Title/Abstract] OR “myocardial failure"[Title/Abstract]
**#3 “**Diastolic"[Title/Abstract] OR “preserved ejection fraction"[Title/Abstract] OR “normal ejection fraction"[Title/Abstract]
**#4** #1 OR #2
**#5** #3 AND #4
**#6 “**Qili Qiangxin"[Title/Abstract] OR “Qishen Yiqi"[Title/Abstract] OR “Yixinshu"[Title/Abstract] OR “Yangxinshi"[Title/Abstract] OR “Shexiang Baoxin"[Title/Abstract] OR “Tongxinluo"[Title/Abstract] OR “Chinese patent medicine"[Title/Abstract]
**#7 “**Tablet"[Title/Abstract] OR “Pill"[Title/Abstract] OR “Capsule"[Title/Abstract] OR “Granule"[Title/Abstract] OR “Powder"[Title/Abstract]
**#8** #6 OR #7
**#9** (“randomized controlled trial"[Publication Type] OR “controlled clinical trial"[Publication Type] OR “randomized"[Title/Abstract] OR “placebo"[Title/Abstract] OR “clinical trials as topic"[MeSH Terms:noexp] OR “randomly"[Title/Abstract] OR “trial"[Title]) NOT (“animals"[MeSH Terms] NOT (“humans"[MeSH Terms] AND “animals"[MeSH Terms]))
**#10** #5 AND #8 AND #9

### Literature inclusion and data extraction

Two researchers (GHX and LXY) independently read and screened the literature according to the inclusion and exclusion criteria and extracted data from the final included literature. The extracted contents mainly included the following: basic information and general characteristics of the included RCTs, information relevant to the literature quality evaluation, intervention control measures, course of treatment, and outcome indicators. After screening the literature and extracting the information, cross-checking was performed. In case of disagreement, consensus was reached with the help of a third author.

### Quality assessment

Two investigators evaluated the methodological quality of the included literature based on seven items (random sequence, allocation concealment, blinding of patients and researchers, blinding of outcome evaluators, integrity of outcome data, selective reporting of results, and other sources of bias) in accordance with the RCT risk-of-bias assessment tool in Cochrane Handbook for Systematic Reviews of Interventions (16). Based on the results, each item was rated as low-risk, unclear-risk, or high-risk. As detailed in Supplementary Material S2.

### Statistical analysis

Bayesian NMA was conducted using a Markov chain Monte Carlo method (17). The initial value was set at 2.5 using four Markov chains. A total of 10,000 preiterations were conducted, followed by 50,000 iterations to achieve model convergence. When the potential scale reduction factor (PSRF) approached 1, the model converged satisfactorily; otherwise, the number of iterations was increased. The evidence network diagram of each result was drawn to visualize the connections between different interventions. When there was a closed loop, inconsistency between the direct and indirect evidence was detected using node splitting. To measure effect size, binary data are expressed as the odds ratio (OR), continuous data are expressed as the weighted mean difference (WMD) with the 95% confidence interval (CI). The random-effects model was used for data merging. *P* < 0.05 was considered statistically significant. The efficacies of different interventions were ranked according to the surface under the cumulative ranking curve (SUCRA) (18). Network meta-regression analysis with year of publication and duration of treatment as covariates was performed to determine whether these factors had any effect on the results. The effect size of multiple outcome indicators was summed, and multidimensional efficacy analysis was performed to identify the best intervention. Publication bias was assessed by funnel plots. The Bayesian NMA was performed using the GeMTC package in R (version 4.1.2).

## Results

### Literature screening results

**Figure 1:**
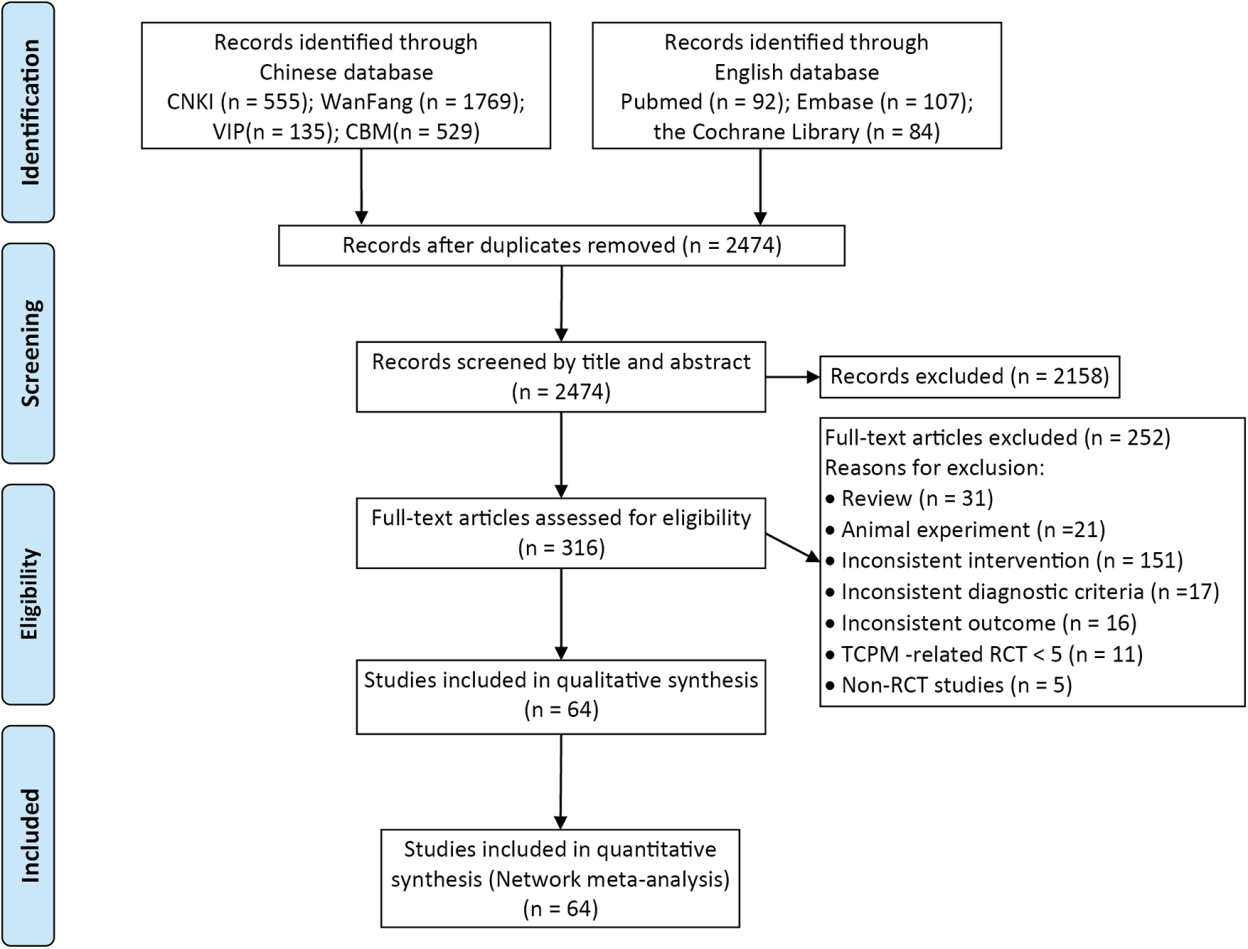
Literature screening flowchart.

A total of 3,271 relevant articles were obtained in the preliminary examination, and 64 RCTs were finally included. The literature search process is shown in Figure 1.

### Basic characteristics of the included literature

Six CTPMs, i.e., Qili Qiangxin capsule (QLQXC), Qishen Yiqi dropping pill (QSYQDP), Yixinshu capsule (YXSC), Yangxinshi tablet (YXST), Shexiang Baoxin Pill (SXBXP), and Tongxinluo capsule (TXLC), were investigated in the 64 RCTs. The 64 RCTs included 6,238 HFPEF patients, CWM treatment was given to the control group, and CWM treatment combined with CTPM treatment was given to the experimental group. The basic characteristics of the included studies are shown in Table 3. Among these RCTs, 27 focused on QLQXC (19, 20, 22, 24, 27–31, 35, 39, 41–43, 46, 50, 53, 54, 59, 61, 63–65, 68, 71, 72, 82), 12 on QSYQDP (21, 32–34, 44, 52, 62, 66, 76, 77, 80, 81), eight on YXSC (38, 40, 45, 48, 55, 57, 73, 79), six on YXST (23, 36, 51, 56, 74, 78), six on SXBXP (26, 37, 49, 58, 70, 75) and five on TXLC (25, 47, 60, 67, 69). The details of the included CTPMs are summarized in Supplementary Material S3, including the source, composition, amount of each component, and extraction procedure of each CTPM, as well as its actions, indications, administration, and chemical analysis.

**Table 3:** Characteristics of the included studies.

References	Cases		Sex (M/F)		Age (years)		Treatment (dosage)		Duration	Outcomes
	T	C	T	C	T	C	T	C	(months)	
Bu and Zhang (19)	30	30	18/12	20/10	69 ± 9	71 ± 9	CWM + QLQXC (1.2 g, tid)	CWM	3	②③④⑤
Chen et al. (20)	102	98	60/42	54/44	58.34 ± 6.68	57.23 ± 6.37	CWM + QLQXC (1.2 g, tid)	CWM	0.5	①③④
Chen et al. (21)	33	33	11/22	18/15	85.06 ± 5.45	86.94 ± 4.25	CWM + QSYQDP (0.5 g, tid)	CWM	3	①③⑤
Chen (22)	33	33	15/18	16/17	58 ± 8.14	59 ± 7.02	CWM + QLQXC (1.2 g, tid)	CWM	2	①②
Cheng et al. (23)	48	48	26/22	25/23	71.88 ± 3.76	71.24 ± 3.59	CWM + YXST (1.2 g, tid)	CWM	1	①②③④⑤
Chi et al. (24)	46	46	50/42		62.1		CWM + QLQXC (1.2 g, tid)	CWM	3	①②
Dai (25)	62	58	31/31	28/30	58.36 ± 5.21	58.37 ± 6.9	CWM + TXLC (1.04 g, tid)	CWM	1	①②
Dong and Wang (26)	56	56	23/33	21/35	75.5 ± 1.2	76.5 ± 1.4	CWM + SXBXP (45 mg, tid)	CWM	1	①④
Dong et al. (27)	35	35	20/15	18/17	68.25 ± 1.23	67.42 ± 1.62	CWM + QLQXC (1.2 g, tid)	CWM	6	②③
Du (28)	51	51	19/32	21/30	60−86	59−82	CWM + QLQXC (1.2 g, tid)	CWM	1	①③⑤
Du (29)	48	47	25/23	25/22	70.67 ± 5.84	68.11 ± 6.20	CWM + QLQXC (1.2 g, tid)	CWM	6	②④
Feng et al. (30)	30	30	14/16	13/17	67.8 ± 19.2	68.6 ± 18.4	CWM + QLQXC (1.2 g, tid)	CWM	3	③
Guan and Yang (31)	42	40	17/25	18/22	56 ± 13	54 ± 13	CWM + QLQXC (1.2 g, tid)	CWM	2	②③④
He (32)	40	40	48/32		56.2		CWM + QSYQDP (0.5 g, tid)	CWM	6	①②⑤
Hou and Yang (33)	60	60	38/22	36/24	61.4 ± 6.21	62.21 ± 7.13	CWM + QSYQDP (0.5 g, tid)	CWM	3	①②③
Hu et al. (34)	40	40	15/25	17/23	67.1 ± 5.2	66.2 ± 6.1	CWM + QSYQDP (0.5 g, tid)	CWM	2	①②⑤
Hu, et al. (35)	42	42	22/20	23/19	58.7 ± 12.4	59.9 ± 11.8	CWM + QLQXC (1.2 g, tid)	CWM	1	①③
Huang et al. (36)	63	62	37/26	36/26	59.8 ± 11.2	61.2 ± 13.4	CWM + YXST (1.2 g, tid)	CWM	1	①⑤
Huang et al. (37)	27	29	20/7	19/10	71.6 ± 9.8	70.5 ± 11.5	CWM + SXBXP (45 mg, tid)	CWM	2	③④
Jian and Ji (38)	58	56	36/22	33/23	64.9 ± 16.8	64.5 ± 15.4	CWM + YXSC (1.2 g, tid)	CWM	6	①②
Jian et al. (39)	39	40	17/22	21/19	67.4 ± 9.2	65.4 ± 10.1	CWM + QLQXC (1.2 g, tid)	CWM	1	①②④⑤
Li and Liu (40)	90	90	43/47	41/49	67.8 ± 11.2	68.6 ± 10.5	CWM + YXSC (1.2 g, tid)	CWM	3	①②④
Li et al. (41)	50	50	21/29	22/28	61.6 ± 5.1	61.4 ± 5.4	CWM + QLQXC (1.2 g, tid)	CWM	6	①③⑤
Li et al. (42)	30	30	21/9	19/11	56.6 ± 8.5	56.9 ± 8.4	CWM + QLQXC (1.2 g, tid)	CWM	3	①②⑤
Li et al. (43)	62	62	34/24	30/32	64.02 ± 5.14	61.24 ± 3.21	CWM + QLQXC (1.2 g, tid)	CWM	1	①②④
Li (44)	40	40	-		62.3 ± 8.6		CWM + QSYQDP (0.5 g, tid)	CWM	3	①②④
Li (45)	30	30	9/21	8/22	70.96 ± 7.66	67.4 ± 9.17	CWM + YXSC (1.2 g, tid)	CWM	2	①③⑤
Li (46)	43	43	23/20	24/19	61.26 ± 4.42	61.19 ± 4.34	CWM + QLQXC (1.2 g, tid)	CWM	2	①
Liao (47)	50	50	28/22	26/24	61.32 ± 9.40	60.26 ± 10.0	CWM + TXLC (0.78 g, tid)	CWM	2	①②
Liu et al. (48)	92	90	-		70.64 ± 9.29	69.29 ± 7.01	CWM + YXSC (1.2 g, tid)	CWM	2	②
Liu et al. (49)	18	17	10/8	12/5	66.83 ± 6.48	64.94 ± 7.64	CWM + SXBXP (45 mg, tid)	CWM	3	④
Liu (50)	30	30	17/13	45,249	62.8 ± 11.3	63.1 ± 10.7	CWM + QLQXC (1.2 g, tid)	CWM	2	②④⑤
Liu (51)	33	32	16/17	14/18	58.39 ± 11.47	57.14 ± 12.81	CWM + YXST (1.2 g, tid)	CWM	3	②③④⑤
Liu (52)	51	51	29/22	28/23	58.67 ± 4.32	59.18 ± 4.46	CWM + QSYQDP (0.5 g, tid)	CWM	3	①②③④
Lu et al. (53)	62	46	28/34	21/25	71.2	70.8	CWM + QLQXC (1.2 g, tid)	CWM	2	①②⑤
Luo and Wan (54)	50	50	23/27	24/26	57.2 ± 8.5	57.4 ± 8.2	CWM + QLQXC (1.2 g, tid)	CWM	6	①②③④⑤
Ma et al. (55)	52	45	28/24	25/20	61.52	63.28	CWM + YXSC (1.2 g, tid)	CWM	3	②④
Pan (56)	80	80	37/43	38/42	60.4 ± 14.8	60.8 ± 14.5	CWM + YXST (1.2 g, tid)	CWM	1	①④
Pei et al. (57)	84	84	52/32	48/36	66.2 ± 4	66.2 ± 3.8	CWM + YXSC (1.2 g, tid)	CWM	3	②
Peng et al. (58)	120	120	62/58	65/55	69.3	67.22	CWM + SXBXP (45 mg, tid)	CWM	24	②⑤
Qiang (59)	44	44	23/21	24/20	57	59	CWM + QLQXC (1.2 g, tid)	CWM	6	③④
Qiu (60)	40	40	17/23	22/18	59.28	59.73	CWM + TXLC (1.04 g, tid)	CWM	1	①
Ruan (61)	100	100	51/49	53/47	61.13 ± 5.78	60.23 ± 6.12	CWM + QLQXC (1.2 g, tid)	CWM	1	②③④
Song (62)	55	55	31/24	30/25	63.6 ± 9.2	64.8 ± 8.9	CWM + QSYQDP (0.5 g, tid)	CWM	2	①②③
Sun (63)	25	25	24/26		59.8 ± 1.4		CWM + QLQXC (1.2 g, tid)	CWM	1	①②⑤
Tian (64)	60	60	28/32	29/31	58 ± 80.2	56 ± 7.8	CWM + QLQXC (1.2 g, tid)	CWM	1	①②⑤
Wang et al. (65)	31	31	20/11	18/13	61.2 ± 1.3	60.7 ± 5.9	CWM + QLQXC (1.2 g, tid)	CWM	6	①③⑤
Wang (66)	52	52	27/25	24/28	70.02 ± 4.03	69.13 ± 4.21	CWM + QSYQDP (0.5 g, tid)	CWM	6	②③
Wei (67)	30	30	13/17	18/12	58.36 ± 5.20	58.38 ± 6.9	CWM + TXLC (1.04 g, tid)	CWM	1	①②⑤
Wu et al. (68)	50	50	27/23	28/22	64.79 ± 7.32	64.32 ± 7.28	CWM + QLQXC (1.2 g, tid)	CWM	3	②
Xu (69)	30	30	16/14	17/13	58.5	60.2	CWM + TXLC (0.78 g, tid)	CWM	6	①②
Yang et al. (70)	23	23	11/12	10/13	48−80		CWM + SXBXP (45 mg, tid)	CWM	3	①
Yang et al. (71)	29	29	15/14	14/15	60.98 ± 4.12	61.23 ± 5.32	CWM + QLQXC (1.2 g, tid)	CWM	6	①③
Yu (72)	35	35	20/15	23/12	65.7 ± 6.1	66.1 ± 8.2	CWM + QLQXC (1.2 g, tid)	CWM	12	①②④⑤
Yu (73)	30	30	18/12	19/11	67.5	68.3	CWM + YXSC (1.2 g, tid)	CWM	3	①④
Yuan (74)	40	35	21/19	18/17	68.7 ± 10.2	71.3 ± 13.1	CWM + YXST (1.2 g, tid)	CWM	1	①④
Zeng and Zhu (75)	56	50	39/17	31/19	51–65	45–70	CWM + SXBXP (45 mg, tid)	CWM	3	②⑤
Zhang and Gou (76)	49	49	28/21	26/23	55.38 ± 5.27	56.72 ± 6.38	CWM + QSYQDP (0.5 g, tid)	CWM	3	①④⑤
Zhang and Li (77)	50	50	20/30	22/38	67 ± 6	68 ± 8	CWM + QSYQDP (0.5 g, tid)	CWM	6	①②④⑤
Zhang and Niu (78)	34	33	18/16	21/12	55.69 ± 9.62	54.05 ± 9.62	CWM + YXST (1.2 g, tid)	CWM	2	①④
Zhang and Zhu (79)	58	52	26/32	24/28	71.1 ± 12.8	69.2 ± 11.4	CWM + YXSC (1.2 g, tid)	CWM	3	①②④
Zhang et al. (80)	90	90	44/46	43/47	70.53 ± 5.28	70.12 ± 6.98	CWM + QSYQDP (0.5 g, tid)	CWM	6	②③④
Zhang et al. (81)	54	54	25/29	22/32	66.28 ± 1.89	66.84 ± 2.57	CWM + QSYQDP (0.5 g, tid)	CWM	3	①③④⑤
Zhen and Tian (82)	30	30	17/13	15/15	62.4 ± 4.4	61.6 ± 4.6	CWM + QLQXC (1.2 g, tid)	CWM	3	②

Age is described as the mean ± standard deviation or mean (minimum—maximum).

T, test group; C, control group; tid, three times a day; CWM, conventional Western medicine; QLQXC, Qili Qiangxin capsule; QSYQDP, Qishen Yiqi dropping pill; YXSC, Yixinshu capsule; YXST Yangxinshi tablet; SXBXP, Shexiang Baoxin pill; TXLC, Tongxinluo capsule. Outcomes: ① New York Heart Association cardiac functional classification efficiency; ② ratio of early diastolic mitral inflow velocity to late diastolic mitral inflow velocity; ③ N-terminal pro-B type natriuretic peptide; ④ 6-minute walking test; ⑤ adverse drug reactions.

### Bias risk assessment

**Figure 2:**
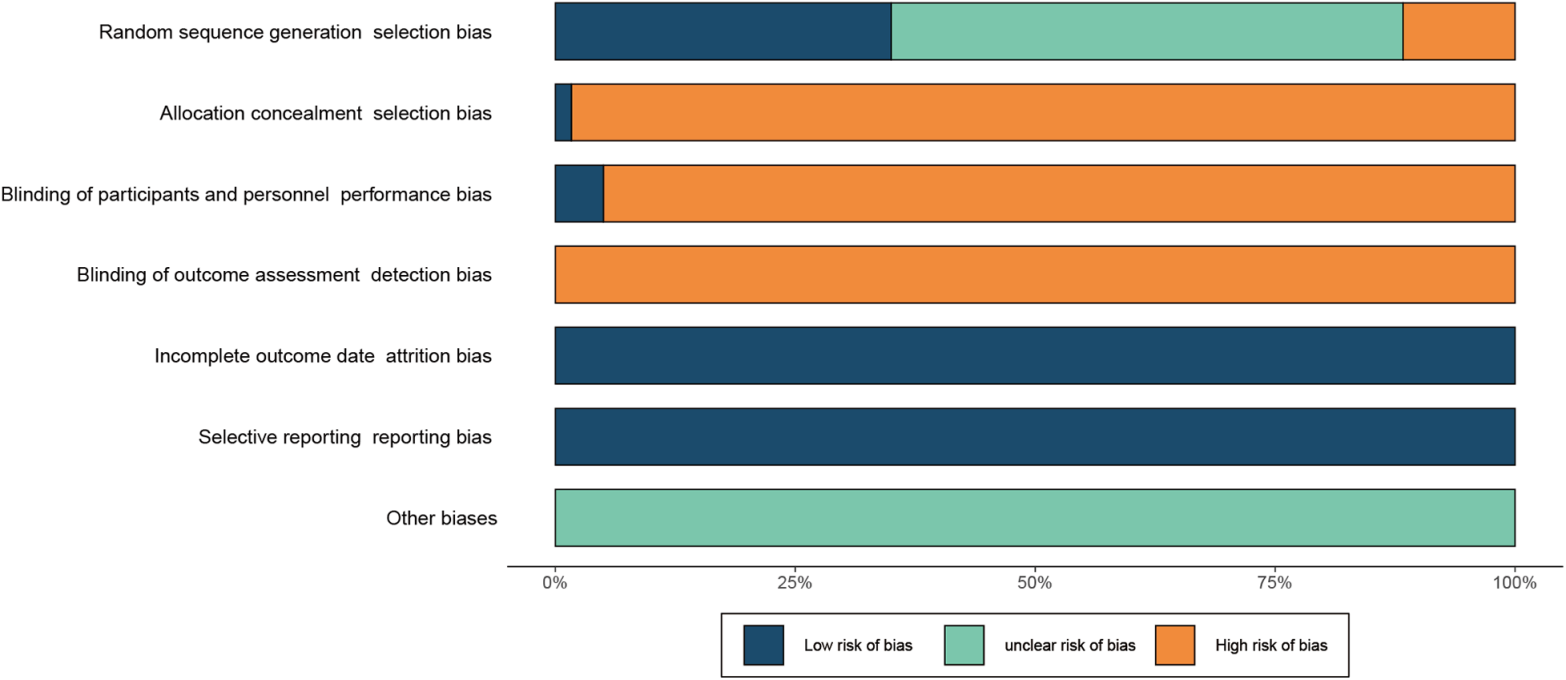
Risk-of-bias summary.

Among the 64 RCTs, 24 (19, 25, 29, 33, 35–37, 46–52, 57, 61, 62, 64, 66, 71, 76, 78–80) used random number tables and were rated as having low risk in the random sequence generation domain. Seven (20, 21, 39, 40, 43, 56, 75) used the order of visits and were rated as having high risk. The other 33 did not describe the random allocation method and were rated as having an unclear risk. One RCT (47) conducted allocation concealment correctly and was rated as having low risk in allocation concealment; the other 63 RCTs did not conduct allocation concealment and thus had high risk. In four RCTs (26, 37, 49, 72), both patients and researchers were blinded and these were rated as having low risk regarding the blinding of patients and researchers. The other 60 RCTs did not blind patients or researchers and were rated as having high risk. None of the RCTs blinded the outcome evaluators and thus had high risk in this domain. All RCTs had complete data and were rated as having low risk of outcome data integrity. None of the RCTs had selective reporting results and were rated as having low risk of selective reporting of results. For all RCTs, the research plans were unavailable, so the risk of other sources of bias was unclear (The risk of bias results are summarized in Figure 2, and the detailed risk of bias assessment results for each study are presented in Supplementary Material S4).

### Results of the Bayesian network meta-analysis

#### NYHA cardiac functional classification efficiency

**Figure 3:**
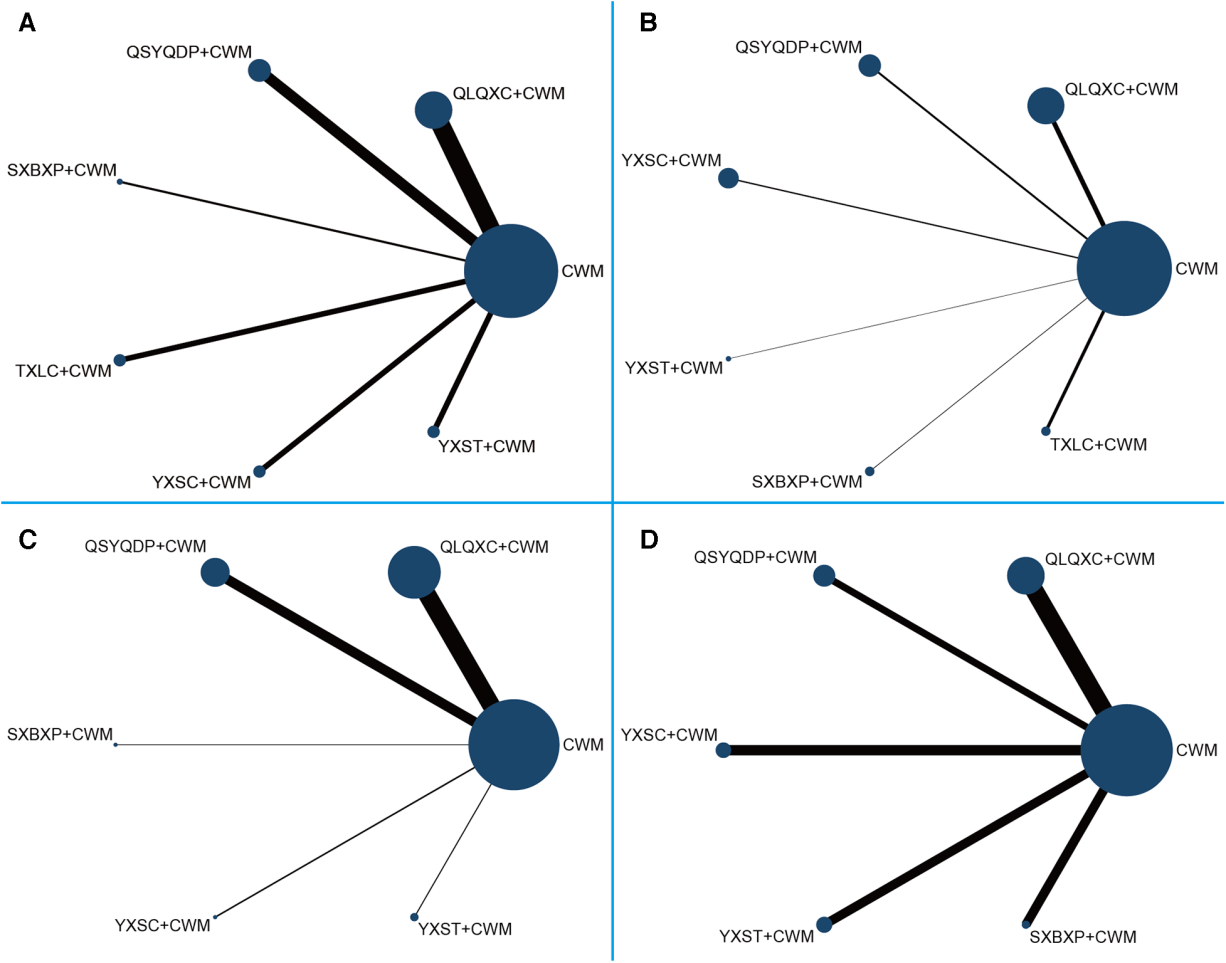
Network meta-analysis of eligible comparisons for different outcomes. (**A**) New York Heart Association cardiac functional classification efficiency; (**B**) the ratio of early diastolic mitral inflow velocity to late diastolic mitral inflow velocity (E/A); (**C**) N-terminal pro-B type natriuretic peptide (NT-proBNP); (**D**) 6-min walking test (6MWT). The size of the nodes relates to the number of participants in that intervention type, and the thickness of lines between the interventions relates to the number of studies for that comparison. CWM, conventional Western medicine; QLQXC, Qili Qiangxin capsule; QSYQDP, Qishen Yiqi dropping pill; YXSC, Yixinshu capsule; YXST Yangxinshi tablet; SXBXP, Shexiang Baoxin pill; TXLC, Tongxinluo capsule.

**Figure 4:**
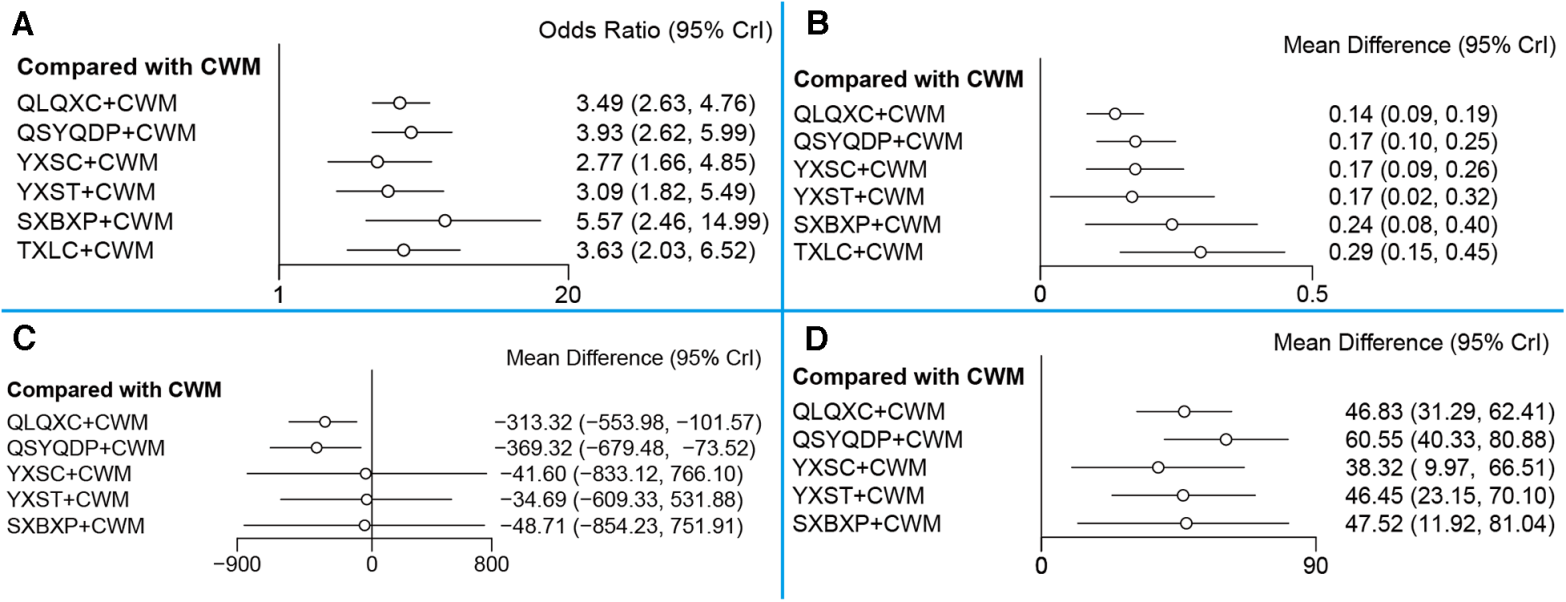
Forest plot for different outcomes. (**A**) New York Heart Association cardiac functional classification efficiency; (**B**) the ratio of early diastolic mitral inflow velocity to late diastolic mitral inflow velocity (E/A); (**C**) N-terminal pro-B type natriuretic peptide (NT-proBNP); (**D**) 6-min walking test (6MWT). CWM, conventional Western medicine; QLQXC, Qili Qiangxin capsule; QSYQDP, Qishen Yiqi dropping pill; YXSC, Yixinshu capsule; YXST Yangxinshi tablet; SXBXP, Shexiang Baoxin pill; TXLC, Tongxinluo capsule.

**Figure 5:**
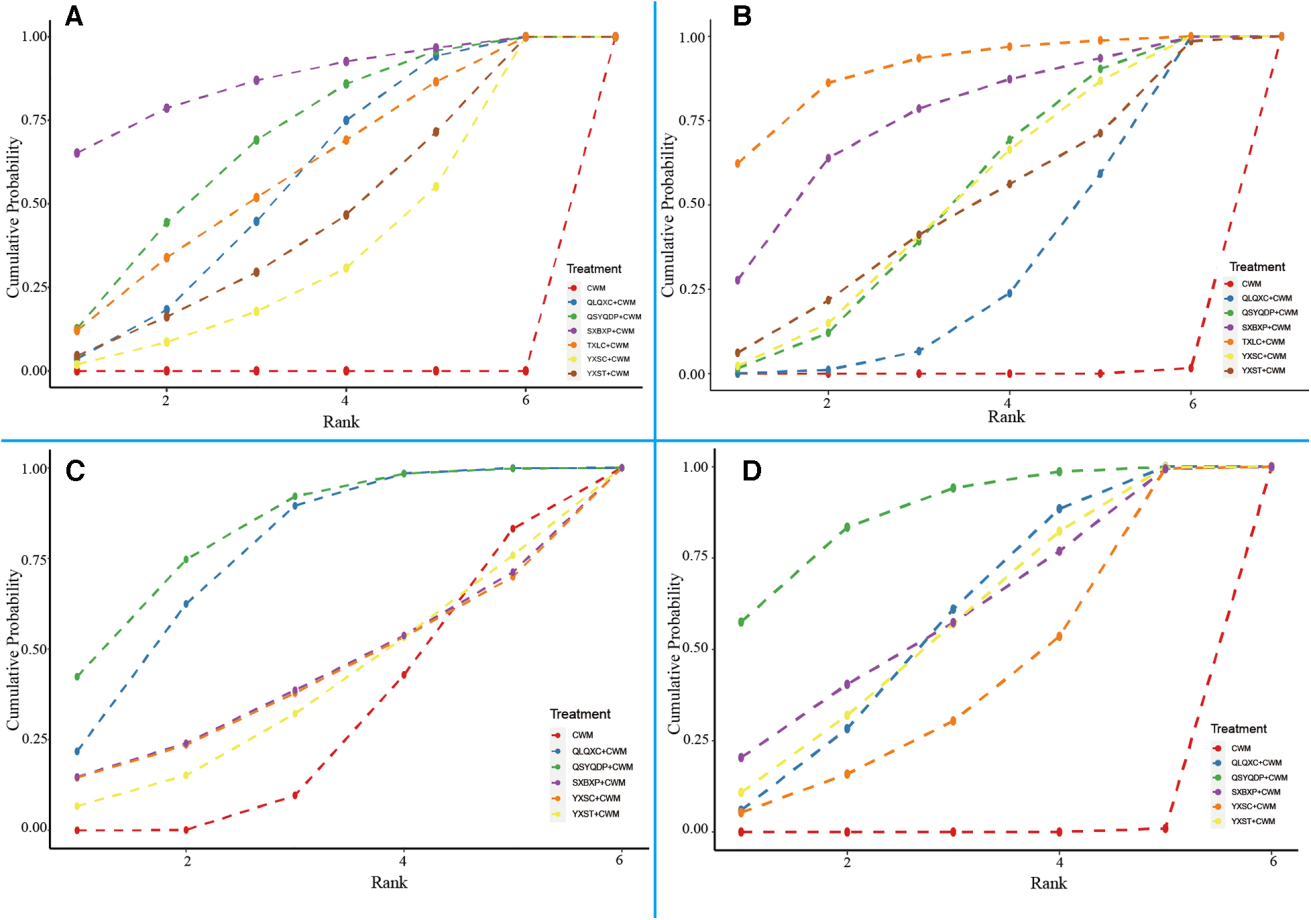
Surface under the cumulative ranking curve (SUCRA) for different outcomes. (**A**) New York Heart Association cardiac functional classification efficiency; (**B**) the ratio of early diastolic mitral inflow velocity to late diastolic mitral inflow velocity (E/A); **(C)** N-terminal pro-B type natriuretic peptide (NT-proBNP); **(D)** 6-min walking test (6MWT). CWM, conventional Western medicine; QLQXC, Qili Qiangxin capsule; QSYQDP, Qishen Yiqi dropping pill; YXSC, Yixinshu capsule; YXST Yangxinshi tablet; SXBXP, Shexiang Baoxin pill; TXLC, Tongxinluo capsule.

A total of 44 RCTs (involving six CTPMs and 4,130 patients) reported NYHA cardiac functional classification efficiency. A network evidence diagram of the interventions is depicted in Figure 3A. The results showed the efficacy of CWM treatment to be significantly lower than that of QLQXC + CWM [OR = 0.29, 95% CI (0.21, 0.38)], QSYQDP + CWM [OR = 0.25, 95% CI (0.17, 0.38)], YXSC + CWM [OR = 0.36, 95% CI (0.21, 0.6)], YXST + CWM [OR = 0.32, 95% CI (0.18, 0.55)], SXBXP + CWM [OR = 0.18, 95% CI (0.07, 0.41)], and TXLC + CWM [OR = 0.28, 95% CI (0.15, 0.49)] (Table 4, Figure 4A). According to SUCRA-based probabilistic ranking results (Table 5, Figure 5A), SXBXP + CWM (86.68%) is likely to be the best intervention for improving NYHA cardiac functional classification efficiency. The ranking of the seven interventions is as follows: SXBXP + CWM > QSYQDP + CWM > TXLC + CWM > QLQXC + CWM > YXST + CWM > YXSC + CWM > CWM.

**Table 4:** Network meta-analysis matrix of results.

Outcomes	CWM	QLQXC + CWM	QSYQDP + CWM	YXSC + CWM	YXST + CWM	SXBXP + CWM	TXLC + CWM
NYHA efficacy	CWM	3.49 (2.63, 4.76)	3.93 (2.62, 5.99)	2.77 (1.66, 4.85)	3.09 (1.82, 5.49)	5.57 (2.46, 14.99)	3.63 (2.03, 6.52)
	0.29 (0.21, 0.38)	QLQXC + CWM	1.12 (0.68, 1.84)	0.79 (0.43, 1.48)	0.89 (0.48, 1.65)	1.6 (0.67, 4.48)	1.03 (0.54, 1.94)
	0.25 (0.17, 0.38)	0.89 (0.54, 1.47)	QSYQDP + CWM	0.71 (0.36, 1.4)	0.79 (0.39, 1.55)	1.42 (0.56, 4.2)	0.92 (0.45, 1.86)
	0.36 (0.21, 0.6)	1.26 (0.68, 2.32)	1.41 (0.71, 2.77)	YXSC + CWM	1.11 (0.52, 2.42)	2.01 (0.75, 6.03)	1.3 (0.58, 2.8)
	0.32 (0.18, 0.55)	1.13 (0.61, 2.1)	1.27 (0.65, 2.55)	0.9 (0.41, 1.91)	YXST + CWM	1.83 (0.64, 5.57)	1.16 (0.53, 2.62)
	0.18 (0.07, 0.41)	0.62 (0.22, 1.48)	0.7 (0.24, 1.8)	0.5 (0.17, 1.33)	0.55 (0.18, 1.56)	SXBXP + CWM	0.65 (0.21, 1.73)
	0.28 (0.15, 0.49)	0.97 (0.52, 1.86)	1.09 (0.54, 2.23)	0.77 (0.36, 1.73)	0.86 (0.38, 1.89)	1.55 (0.58, 4.73)	TXLC + CWM
E/A	CWM	0.14 (0.09, 0.19)	0.17 (0.10, 0.25)	0.17 (0.08, 0.26)	0.17 (0.02, 0.32)	0.24 (0.08, 0.4)	0.29 (0.15, 0.45)
	−0.14 (−0.19, −0.09)	QLQXC + CWM	0.04 (−0.05, 0.13)	0.04 (−0.06, 0.14)	0.03 (−0.13, 0.19)	0.1 (−0.06, 0.27)	0.16 (0, 0.32)
	−0.17 (−0.25, −0.1)	−0.04 (−0.13, 0.05)	QSYQDP + CWM	0 (−0.12, 0.11)	−0.01 (−0.17, 0.16)	0.07 (−0.11, 0.24)	0.12 (−0.04, 0.29)
	−0.17 (−0.26, −0.08)	−0.04 (−0.14, 0.06)	0 (−0.11, 0.12)	YXSC + CWM	−0.01 (−0.18, 0.17)	0.07 (−0.11, 0.25)	0.12 (−0.05, 0.3)
	−0.17 (−0.32, −0.02)	−0.03 (−0.19, 0.13)	0.01 (−0.16, 0.17)	0.01 (−0.17, 0.18)	YXST + CWM	0.07 (−0.15, 0.29)	0.13 (−0.08, 0.34)
	−0.24 (−0.4, −0.08)	−0.1 (−0.27, 0.06)	−0.07 (−0.24, 0.11)	−0.07 (−0.25, 0.11)	−0.07 (−0.29, 0.15)	SXBXP + CWM	0.05 (−0.16, 0.27)
	−0.29 (−0.45, −0.15)	−0.16 (−0.32, 0)	−0.12 (−0.29, 0.04)	−0.12 (−0.3, 0.05)	−0.13 (−0.34, 0.08)	−0.05 (−0.27, 0.16)	TXLC + CWM
NT-proBNP (pg/ml)	CWM	−313.32 (−553.98, −101.57)	−369.32 (−679.48, −73.52)	−41.6 (−833.12, 766.1)	−34.69 (−609.33, 531.88)	−48.71 (−854.23, 751.91)	
	313.32 (101.57, 553.98)	QLQXC + CWM	−55.66 (−423.9, 329.32)	272.95 (−547.57, 1,134.13)	281.44 (−323.98, 909.14)	264.24 (−556.53, 1,117.3)	
	369.32 (73.52, 679.48)	55.66 (−329.32, 423.9)	QSYQDP + CWM	326.95 (−520.16, 1,205.1)	336.06 (−315.62, 985.1)	317.31 (−537.44, 1,196.06)	
	41.60 (−766.1, 833.12)	−272.95 (−1,134.13, 547.57)	−326.95 (−1,205.1, 520.16)	YXSC + CWM	8.71 (−991.85, 994.36)	−9.19 (−1,154.23, 1,109.32)	
	34.69 (−531.88, 609.33)	−281.44 (−909.14, 323.98)	−336.06 (−985.1, 315.62)	−8.71 (−994.36, 991.85)	YXST + CWM	−13.89 (−986.73, 970.28)	
	48.71 (−751.91, 854.23)	−264.24 (−1,117.3, 556.53)	−317.31 (−1,196.06, 537.44)	9.19 (−1,109.32, 1,154.23)	13.89 (−970.28, 986.73)	SXBXP + CWM	
6MWT (m)	CWM	46.83 (31.29, 62.41)	60.55 (40.33, 80.88)	38.32 (9.97, 66.51)	46.45 (23.15, 70.1)	47.52 (11.92, 81.04)	
	−46.83 (−62.41, −31.29)	QLQXC + CWM	13.6 (−11.93, 39.24)	−8.6 (−41.24, 23.9)	−0.36 (−28.5, 27.84)	0.65 (−37.84, 37.45)	
	−60.55 (−80.88, −40.33)	−13.6 (−39.24, 11.93)	QSYQDP + CWM	−22.26 (−56.97, 12.47)	−14.09 (−44.83, 17.29)	−13.12 (−53.96, 25.88)	
	−38.32 (−66.51, −9.97)	8.6 (−23.9, 41.24)	22.26 (−12.47, 56.97)	YXSC + CWM	8.25 (−28, 44.32)	9.02 (−36.88, 52.64)	
	−46.45 (−70.1, −23.15)	0.36 (−27.84, 28.5)	14.09 (−17.29, 44.83)	−8.25 (−44.32, 28)	YXST + CWM	1.12 (−42.16, 41.39)	
	−47.52 (−81.04, −11.92)	−0.65 (−37.45, 37.84)	13.12 (−25.88, 53.96)	−9.02 (−52.64, 36.88)	−1.12 (−41.39, 42.16)	SXBXP + CWM	

All data are given as odds ratio or weighted mean difference (95% confidence interval).

NYHA efficacy, New York Heart Association cardiac functional classification efficiency; E/A, the ratio of early diastolic mitral inflow velocity to late diastolic mitral inflow velocity; NT-proBNP, N-terminal pro-B type natriuretic peptide; 6MWT, 6-minute walking test; OR, odds ratio; WMD, weight mean difference; CI; CWM, conventional Western medicine; QLQXC, Qili Qiangxin capsule; QSYQDP, Qishen Yiqi dropping pill; YXSC, Yixinshu capsule; YXST Yangxinshi tablet; SXBXP, Shexiang Baoxin pill; TXLC, Tongxinluo capsule.

**Table 5:** Results of the surface under the cumulative ranking curve for different outcomes.

Outcomes	CWM	QLQXC + CWM	QSYQDP + CWM	YXSC + CWM	YXST + CWM	SXBXP + CWM	TXLC + CWM
NYHA efficacy	0.00	55.98	67.97	35.71	44.74	86.68	58.91
E/A	0.28	31.82	52.09	51.86	49.20	75.12	89.63
NT-proBNP	27.16	74.43	81.49	39.82	36.63	40.46	
6MWT	0.19	56.77	86.76	40.95	56.43	58.90	

All data are given as percentages. Red and orange indicate the most and second-most likely to be the best intervention to improve this outcome, respectively.

NYHA efficacy, New York Heart Association cardiac functional classification efficiency; E/A, the ratio of early diastolic mitral inflow velocity to late diastolic mitral inflow velocity; NT-proBNP, N-terminal pro-B type natriuretic peptide; 6MWT, 6-minute walking test; CWM, conventional Western medicine; QLQXC, Qili Qiangxin capsule; QSYQDP, Qishen Yiqi dropping pill; YXSC, Yixinshu capsule; YXST Yangxinshi tablet; SXBXP, Shexiang Baoxin pill; TXLC, Tongxinluo capsule.

#### E/A

A total of 41 RCTs (involving six CTPMs and a total of 4,235 patients) reported E/A. The network evidence diagram of the interventions is illustrated in Figure 3B. The results indicated that E/A after CWM treatment is significantly worse than that after QLQXC + CWM [WMD = −0.14, 95% CI (−0.19, −0.09)], QSYQDP + CWM [WMD = −0.17, 95% CI (−0.25, −0.1)], YXSC + CWM [WMD = −0.17, 95% CI (−0.26, −0.08)], YXST + CWM [WMD = −0.17, 95% CI (−0.32, −0.02)], SXBXP + CWM [WMD = −0.24, 95% CI (−0.40, −0.08)], and TXLC + CWM [WMD = −0.29, 95% CI (−0.45, −0.15)] (Table 4, Figure 4B). The SUCRA-based probability ranking results (Table 5, Figure 5B) indicated that TXLC + CWM (89.63%) is likely to be the best intervention to improve E/A. The ranking of the seven interventions is as follows: TXLC + CWM > SXBXP + CWM > QSYQDP + CWM > YXSC + CWM > YXST + CWM > QLQXC + CWM > CWM.

#### NT-proBNP

A total of 24 RCTs (involving five CTPMs and a total of 2,322 patients) reported NT-proBNP. A network evidence diagram of the interventions is shown in Figure 3C. Based on the results, the NT-proBNP concentration after CWM treatment was significantly higher than that after QLQXC + CWM [WMD = 313.32, 95% CI (101.57, 553.98)] or QSYQDP + CWM [WMD = 369.32, 95% CI (73.52, 679.48)] but was not significantly different from that after YXSC + CWM [WMD = 41.60, 95% CI (−766.1, 833.12)], YXST + CWM [WMD = 34.69, 95% CI (−531.88, 609.33)], or SXBXP + CWM [WMD = 48.71, 95% CI (−751.91, 854.23)] (Table 4, Figure 4C). The SUCRA-based probabilistic ranking results (Table 5, Figure 5C) indicated that QSYQDP + CWM (81.49%) is likely to be the best intervention to reduce NT-proBNP. The ranking of the six interventions is as follows: QSYQDP + CWM > QLQXC + CWM > SXBXP + CWM > YXSC + CWM > YXST + CWM > CWM.

#### 6MWT

A total of 29 RCTs (involving five CTPMs and a total of 2,924 patients) reported 6MWT. A network evidence diagram of the interventions is shown in Figure 3D. The 6MWT after CWM treatment was significantly worse than that after QLQXC + CWM [WMD = −46.83, 95% CI (−62.41, −31.29)], QSYQDP + CWM [WMD = −60.55, 95% CI (−80.88, −40.33)], YXSC + CWM [WMD = −38.32, 95% CI (−66.51, −9.97)], YXST + CWM [WMD = −46.45, 95% CI (−70.01, −23.15)], or SXBXP + CWM [WMD = −47.52, 95% CI (−81.04, −11.92)] (Table 4, Figure 4D). The SUCRA-based probabilistic ranking results (Table 5, Figure 5D) indicated that QSYQDP + CWM (86.76%) is likely to be the best intervention for improving the 6MWT. The ranking of the six interventions is as follows: QSYQDP + CWM > SXBXP + CWM > QLQXC + CWM > YXST + CWM > YXSC + CWM > CWM.

#### Safety

Overall, the six CTPMs caused ADRs in a total of 25 RCTs. The reported ADRs were mainly diarrhea, nausea, dry mouth, headache, and fatigue, with no significant differences in incidence between the experimental and control groups. These results demonstrated the six CTPMs to be relatively safe. Because the criteria for the determination of ADRs were different in each RCT, the incidence of ADRs was not assessed via NMA in this study.

#### Network meta-regression analysis

**Figure 6:**
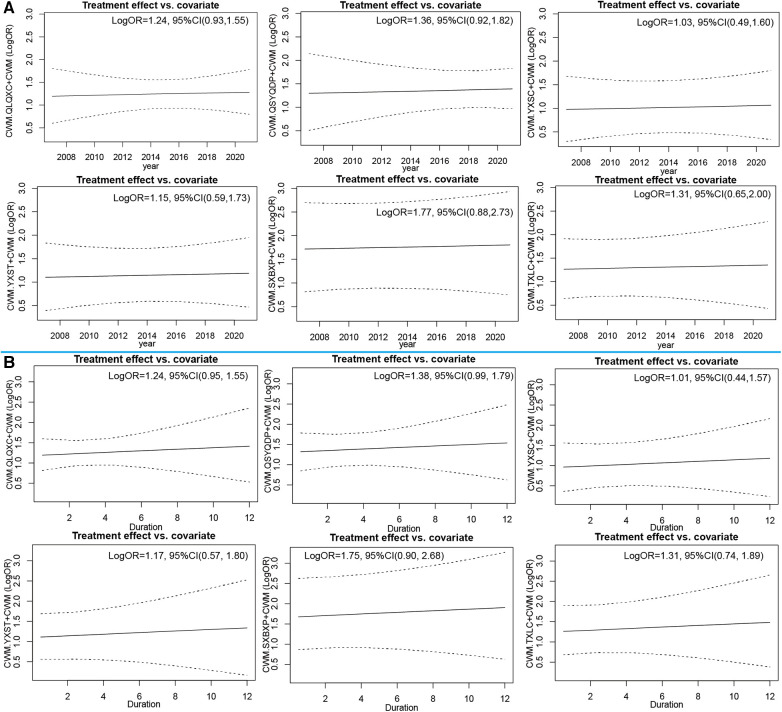
Network meta-regression analysis for New York Heart Association cardiac functional classification efficiency. (**A**) year of publication (**B**) duration of the intervention as covariate.CWM, conventional Western medicine; QLQXC, Qili Qiangxin capsule; QSYQDP, Qishen Yiqi dropping pill; YXSC, Yixinshu capsule; YXST Yangxinshi tablet; SXBXP, Shexiang Baoxin pill; TXLC, Tongxinluo capsule.

Regression analyses were conducted on NYHA efficiency with year of publication and treatment duration as covariates to determine whether these covariates had an effect on the outcome. Before regression, the logORs for interventions QLQXC + CWM, QSYQDP + CWM, YXSC + CWM, YXST + CWM, SXBXP + CWM, and TXLC + CWM compared to CWM were 1.8, 1.97, 1.47, 1.63, 2.48, and 1.86, respectively. The regression-adjusted logORs all decreased, but their 95% confidence intervals (CIs) were all >0, indicating that the CTPMs + CWM treatments were still superior to CWM alone after adjusting. The rankings of the adjusted logOR for each intervention did not change significantly from the pre-adjustment. Simultaneously, the regression coefficient B for year as a covariate was 0.05 (95% CI: −0.45, 0.54), and the regression coefficient B for treatment duration as a covariate was 0.09 (95% CI: −0.34, 0.53), neither of which reached statistical significance (*P* ≥ 0.05), indicating that the covariates had a minimal impact on each intervention. See Figure 6 for details.

#### Consistency tests

Figure 3 reveals no closed loops between these interventions, and a consistency test was thus unnecessary.

#### Model convergence evaluation

**Figure 7:**
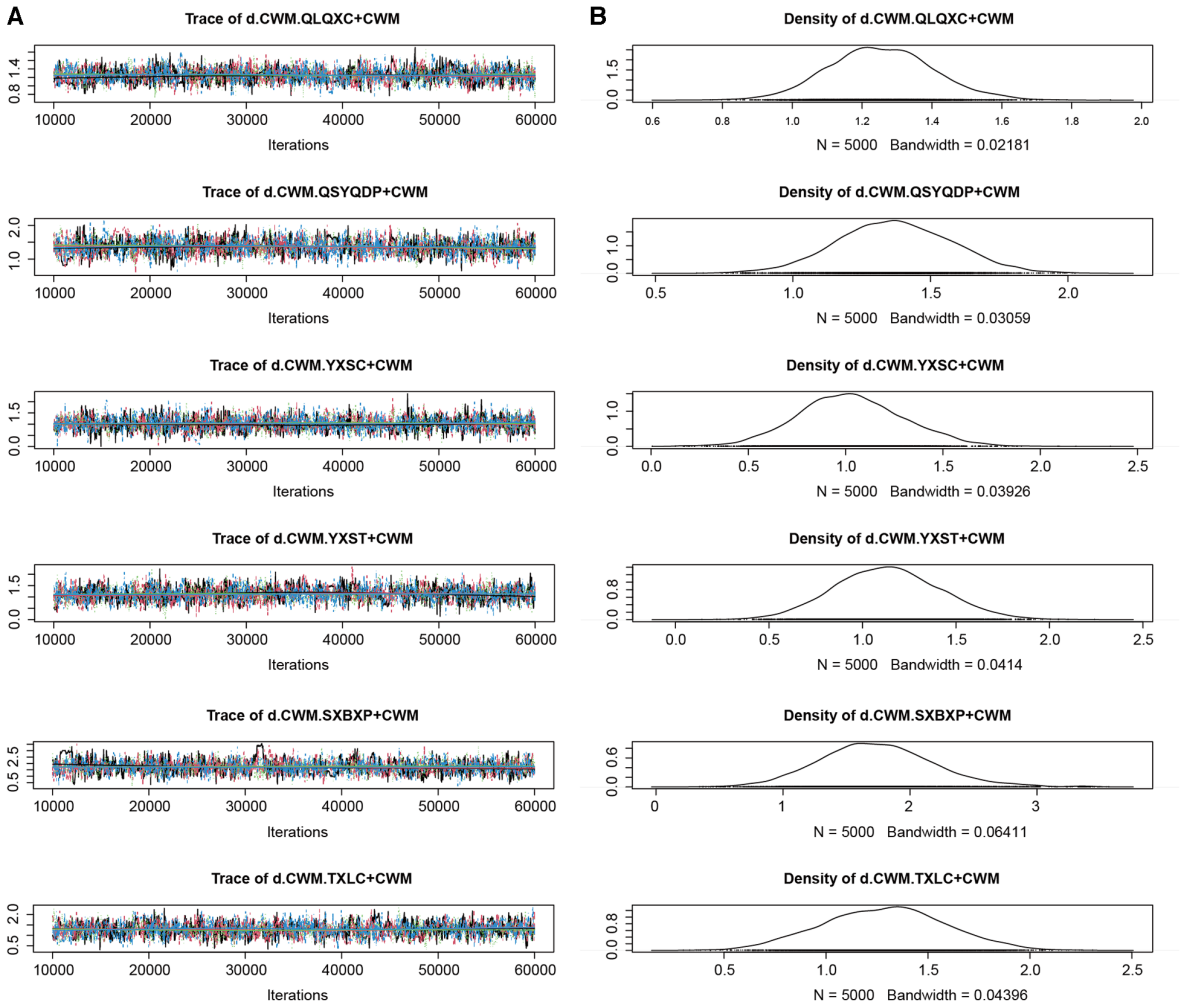
Trace plot and density plot for New York Heart Association cardiac functional classification efficiency. (**A**) Trace plot; (**B**) density plot. CWM, conventional Western medicine; QLQXC, Qili Qiangxin capsule; QSYQDP, Qishen Yiqi dropping pill; YXSC, Yixinshu capsule; YXST Yangxinshi tablet; SXBXP, Shexiang Baoxin pill; TXLC, Tongxinluo capsule.

**Figure 8:**
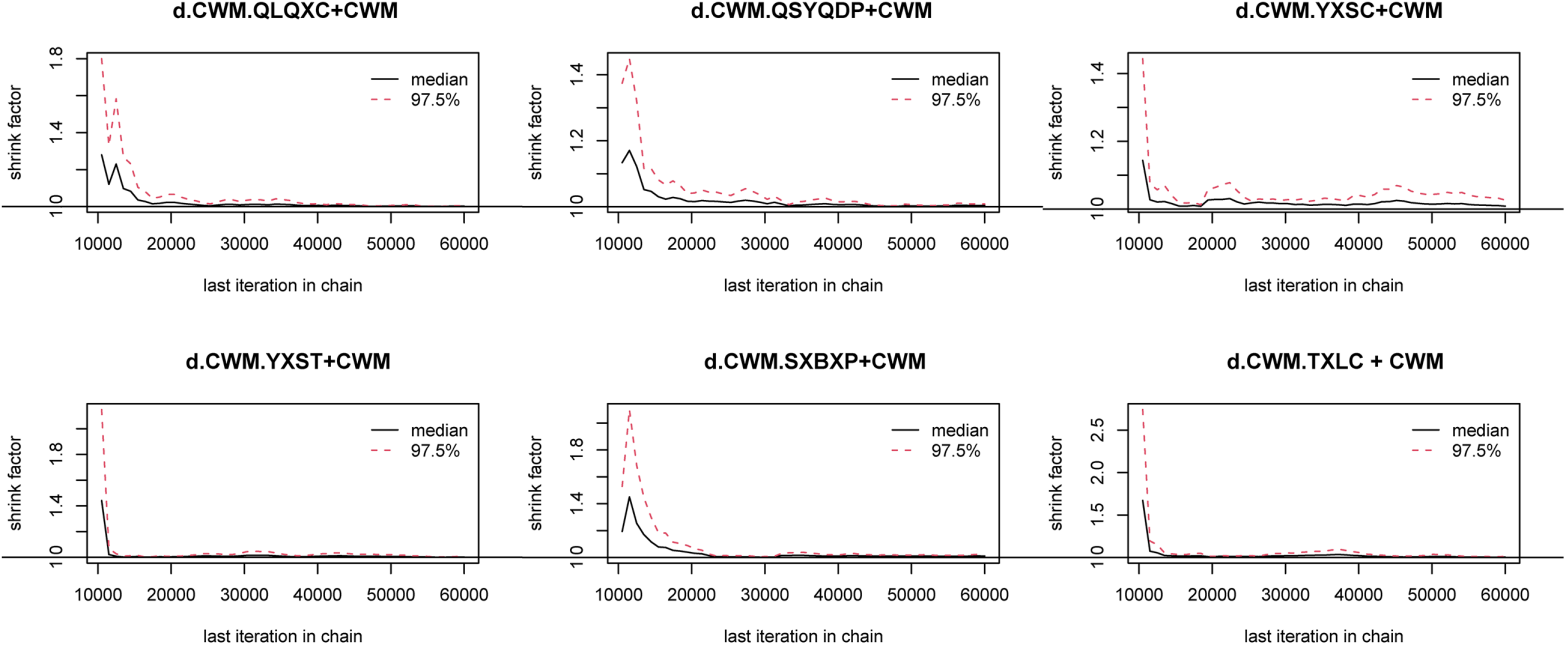
Brooks-Gelman-Rubin diagnosis plot for New York Heart Association cardiac functional classification efficiency. CWM, conventional Western medicine; QLQXC, Qili Qiangxin capsule; QSYQDP, Qishen Yiqi dropping pill; YXSC, Yixinshu capsule; YXST Yangxinshi tablet; SXBXP, Shexiang Baoxin pill; TXLC, Tongxinluo capsule.

In the Bayesian model constructed in this study, each Markov chain in the trace diagrams of the NYHA cardiac functional classification efficiency (an outcome index) in the iterative process (Figure 7A) reached stable convergence from the beginning. In addition, the overlap in the subsequent calculations accounted for most of the fluctuation in the chain, without no fluctuations in a single chain identifiable by the naked eye, indicating satisfactory convergence. Densities (Figure 7B) followed approximately normal distributions, with small preset bandwidths, indicating satisfactory model convergence. The Gelman-Rubin-Brooks plot (Figure 8) showed that after 10,000 preiterations, the median and 97.5th-percentile value of the shrink factor rapidly approached 1 and stabilized, with a PSRF of 1, indicating satisfactory model convergence. The results on model convergence for the other outcome indices are provided in Supplementary Material S5. The results all suggest satisfactory model convergence and sufficient iterations. Therefore, the Bayesian model constructed in this study can effectively predict the posterior distribution of the parameters.

#### Publication bias

**Figure 9:**
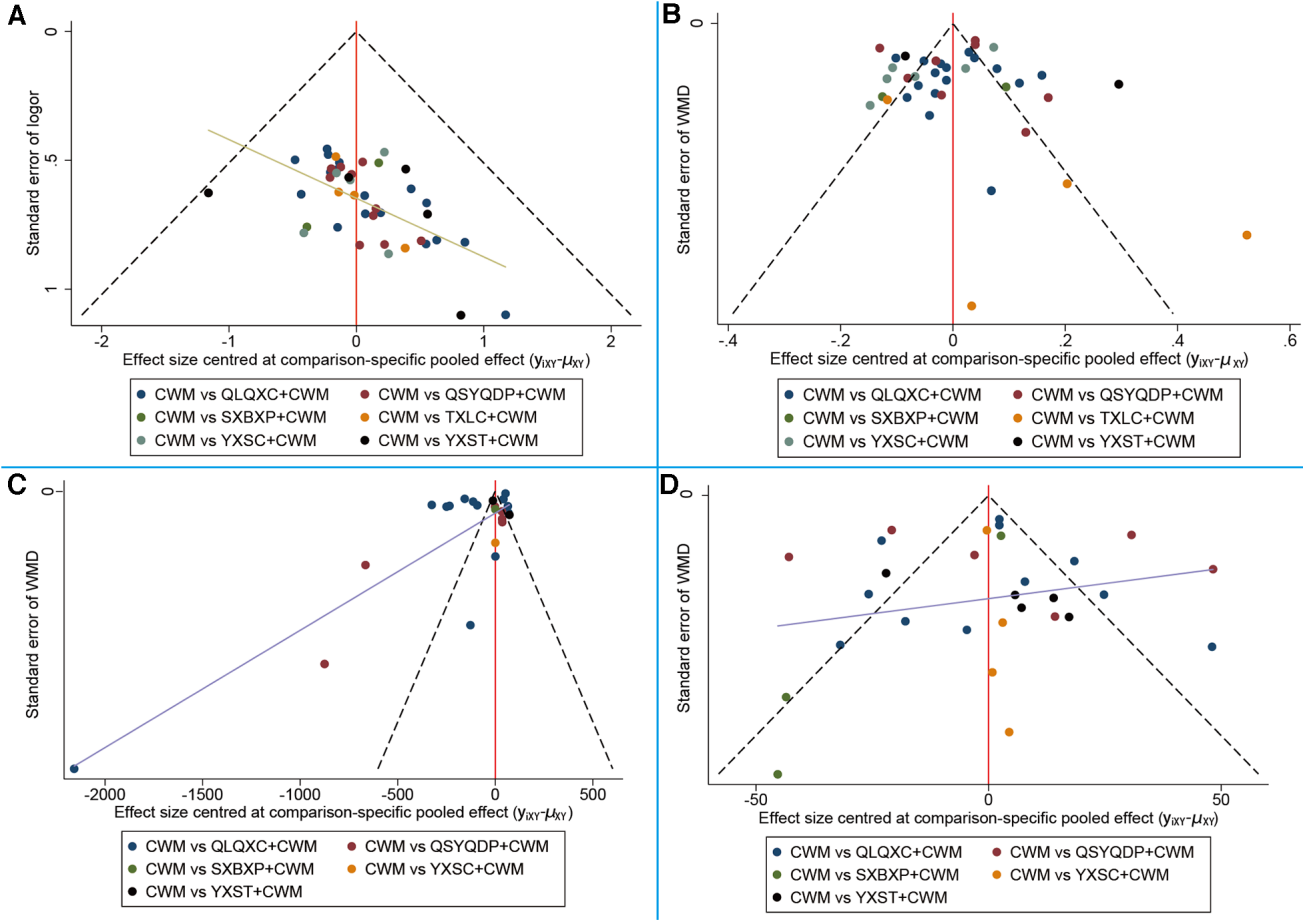
Comparison-adjusted funnel plot for different outcomes. (**A**) New York Heart Association cardiac functional classification efficiency; (**B**) the ratio of early diastolic mitral inflow velocity to late diastolic mitral inflow velocity (E/A); (**C**) N-terminal pro-B type natriuretic peptide (NT-proBNP); (**D**) 6-min walking test (6MWT). CWM, conventional Western medicine; QLQXC, Qili Qiangxin capsule; QSYQDP, Qishen Yiqi dropping pill; YXSC, Yixinshu capsule; YXST Yangxinshi tablet; SXBXP, Shexiang Baoxin pill; TXLC, Tongxinluo capsule.

Comparison-adjusted funnel plots were drawn to assess the publication bias of each outcome indicator (see Figure 9). Each point on the funnel plots represents an RCT. The points in Figures 9A,B,D are roughly symmetrically distributed, indicating that the likelihood of publication bias in the NYHA cardiac functional classification efficiency, E/A, and 6MWT was small. The points in Figure 9C are asymmetrically distributed, indicating that the NT-proBNP results may include publication bias.

#### Comprehensive analysis of multiple outcome indicators

**Figure 10:**
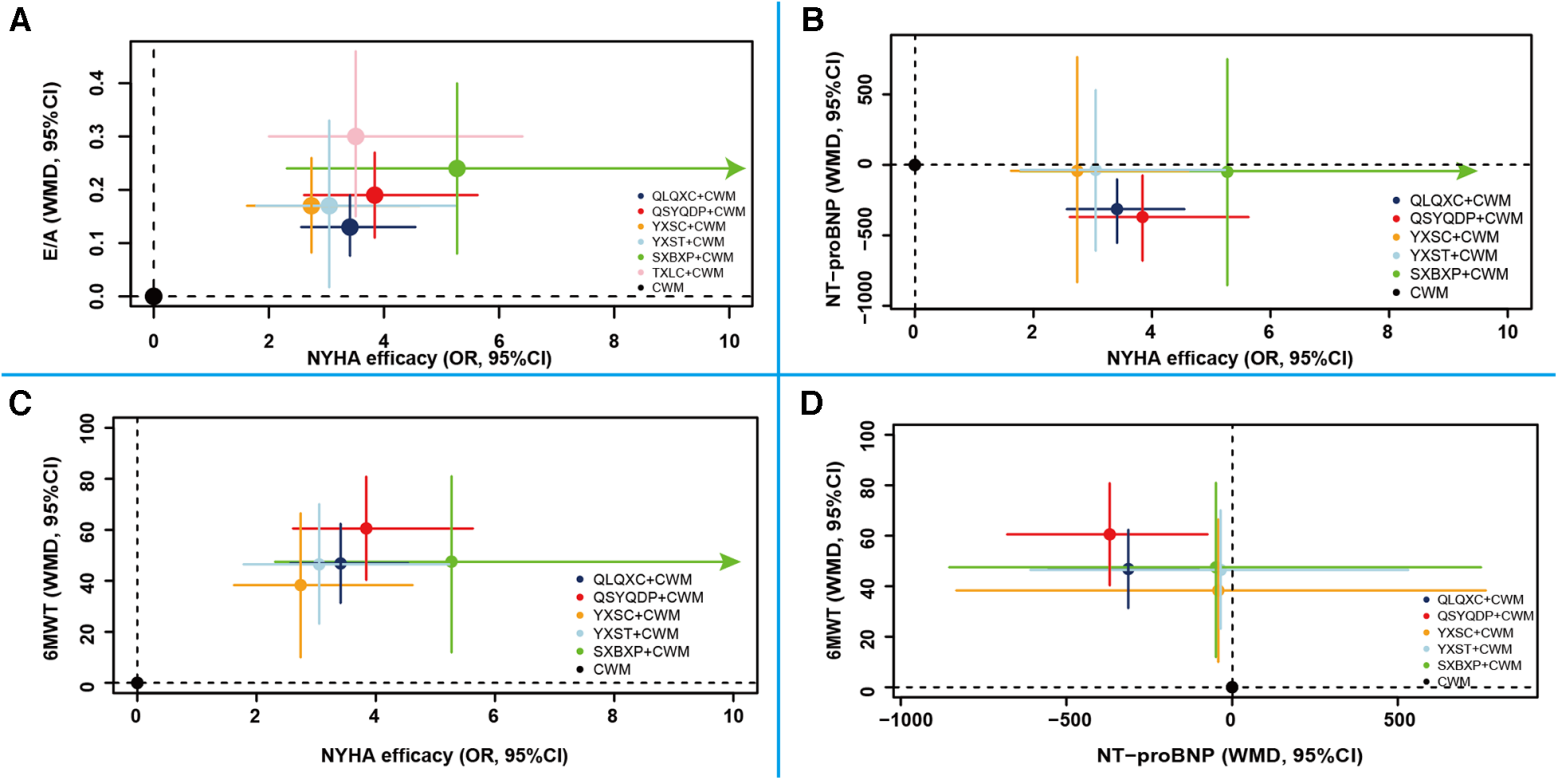
Comprehensive analysis of multiple outcome indicators. Points of different colors represent different interventions, and solid lines of different colors indicate 95% confidence intervals for the effects of the interventions on different outcomes. (**A**) New York Heart Association cardiac functional classification efficiency and E/A; (**B**) New York Heart Association cardiac functional classification efficiency and NT−proBNP; (**C**) New York Heart Association cardiac functional classification efficiency and 6MWT; (**D**) NT-proBNP and 6MWT. NYHA efficacy, New York Heart Association functional classification improvement; E/A, the ratio of early diastolic mitral inflow velocity to late diastolic mitral inflow velocity; NT-proBNP, N-terminal pro-B type natriuretic peptide; 6MWT, 6-min walking test; CWM, conventional Western medicine; QLQXC, Qili Qiangxin capsule; QSYQDP, Qishen Yiqi dropping pill; YXSC, Yixinshu capsule; YXST Yangxinshi tablet; SXBXP, Shexiang Baoxin pill; TXLC, Tongxinluo capsule.

This study included four outcome indicators for efficacy evaluation, which were comprehensively analyzed to find the best interventions for each. Figure 10A shows that SXBXP + CWM, QSYQDP + CWM, and TXLC + CWM may be the best interventions for improving NYHA cardiac functional classification efficiency and E/A. Figure 10B shows that QSYQDP + CWM and SXBXP + CWM may be the best interventions for improving NYHA cardiac functional classification efficiency. Figure 10C shows that QSYQDP + CWM and SXBXP + CWM may be the best interventions for improving NYHA cardiac functional classification efficiency and 6MWT. Figure 10D shows that QSYQDP + CWM may be the best intervention for reducing NT-proBNP and improving 6MWT. In summary, QSYQDP + CWM and SXBXP + CWM may be the best interventions for HFpEF.

## Discussion

HFpEF is associated with substantial morbidity and mortality, and modern medicine offers less effective treatment (83). Various CTPMs have shown good efficacy in the context of HFpEF, but the similarities and differences in their functionality are not clear (84). Therefore, selecting the most suitable CTPMs for patients has become an important clinical problem that needs to be solved. Bayesian NMA can utilize posterior probability based on prior distribution to rank the efficacy of all interventions involved in the estimation. It overcomes the limitations of the frequentist method in parameter estimation, which requires iterative estimation of the likelihood function and is susceptible to instability, leading to biased results (85). In light of these considerations, this study conducted a comprehensive search for RCTs on CTPMs for HFpEF to indirectly compare the efficacies of different interventions for HFpEF and identify the best treatment plan using Bayesian NMA. Ultimately, six CTPMs (QLQXC, QSYQDP, YXSC, YXST, SXBXP, and TXLC) studied in 64 RCTs involving 6,238 patients were included in this NMA. SXBXP + CWM demonstrated the highest efficiency in improving NYHA cardiac functional classification, TXLC + CWM was the most effective at improving E/A, and QSYQDP + CWM provided the greatest advantage in reducing NT-proBNP levels and improving 6MWT distance. Among the SUCRA-based rankings for all outcome indicators, CWM alone ranked low, indicating that integrative treatment is superior to CWM alone for HFpEF. The ADR rates associated with CWM + CTPMs were not significantly different from those associated with CWM alone, suggesting that these CTPMs have good safety. Network meta-regression analysis suggested that year of publication and duration of treatment had a minimal effect on the results. Publication bias analysis indicated possible publication bias for NT-proBNP, which may be attributable to the substantial heterogeneity among the included RCTs or the small sample size of a few of them. A comprehensive analysis of all outcome indicators revealed that QSYQDP + CWM and SXBXP + CWM may be the most favorable treatment options for HFpEF.

Previous reviews have reported that various TCM preparations, including decoctions, powders, tablets, capsules, and others, have shown positive therapeutic effects on HFpEF (84, 86). However, these reviews have described these effects without quantitative analysis. Moreover, many of these preparations, particularly granules, are currently in the clinical trial stage and have not yet received approval from the NMPA of China, which limits their clinical promotion. There have been several systematic quantitative evaluation studies of CTPMs for the treatment of HFPEF, and these CTPMs are approved by the NMPA of China. Wang et al. (9) conducted a systematic review that demonstrated the improvement in NYHA efficiency, E/A, 6MWT, and the reduction in BNP when using QSYQDP + CWM compared to CWM alone. Cao et al. (11) and Ge et al. (87) found that QLQXC + CWM improved clinical efficiency, E/A, 6MWT and reduced BNP, NT-proBNP and Minnesota living with heart failure questionnaire scores compared to CWM. These meta-analyses were all based on single CTPM studies, and their results align with some of the results from this NMA. Jun et al. (10) included multiple CTPMs and found that combining CTPMs with CWM for HFPEF was more advantageous than using CWM alone, resulting in improved efficiency, E/A, 6MWT, and reduced BNP. However, the literature included in this study was not comprehensive enough, and no subgroup analysis was conducted to rank the efficacy of different CTPMs (10). This NMA overcame the shortcomings of the aforementioned studies and obtained results consistent with previous findings, as well as new insights. The NMA results indicate that different CTPMs offer varying advantages in treating HFpEF, with QSYQDP + CWM and SXBXP + CWM standing out as the most prominent options.

In 2016, *Expert consensus on the diagnosis and treatment of chronic heart failure with Integrative Medicine* published by the Chinese Association of Integrative Medicine noted that HF is a syndrome of root vacuity and tip repletion and that the pathogenesis of HF can be summarized as “deficiency”, “stasis”, and “edema” (88). Root vacuity refers to a deficiency in qi, yin and yang. Tip repletion refers to blood stasis, phlegm turbidity, and excessive fluid. Therefore, the basic treatment methods for HF include replenishing qi, promoting blood circulation, and inducing diuresis. As a type of HF, the basic pathogenesis of HFPEF has its own characteristics. A big data study reported that the pathogenesis of HFPEF is characterized by “qi deficiency”, followed by “blood stasis” but that, yang deficiency, yin deficiency, retained fluid and phlegm turbidity do not play a significant role in its development (89). Expert consensus on staging diagnosis combining Chinese and Western medicine in HFPEF states that the pathogenesis of HFPEF is different from that of HFrEF: inhibited qi transformation of heart-lung is predominant in the early stage, accompanied by static blood and retained fluid, resulting in lung failing to diffuse and govern descent; in the middle stage, the mechanism is dominated by inhibited qi transformation of the heart-lung-spleen, with gradually aggravating qi deficiency, or accompanied by yang deficiency, with static blood and retained fluid further aggravated compared with the early stage; in the late stage, the pathogenesis is dominated by inhibited qi transformation of heart-lung-spleen-kidney, yang deficiency has become a reality, and water-dampness is overflowing, or yin deficiency may be seen at the same time (90). The 2022 TCM guidelines for chronic heart failure pointed out that the three main TCM patterns for HFPEF are qi deficiency and blood stasis pattern, qi-yin deficiency and blood stasis pattern, and yang deficiency and blood stasis pattern (91). The six CTPMs included in this study have both similarities and differences in functionality. QLQXC can replenish qi, warm yang, activate blood, unblock collaterals, and induce diuresis to alleviate edema, so it is recommended for yang deficiency, obstruction of collaterals and water retention pattern. QSYQDP is suitable for qi deficiency and blood stasis syndrome as it replenishes qi, frees vessels, and activates blood to relieve pain. YXSC is recommended for the pattern of dual deficiency of qi and yin, or static blood obstructing collaterals as it replenishes qi, restores pulse, activates blood, resolves stasis, nourishes yin, and engenders fluid. YXST is suitable for treating qi deficiency with blood stasis syndrome as it can replenish qi, activate blood, resolve stasis, and relieve pain. SXBXP can warm and unblock meridians with aromatic medicinals, replenish qi and strengthen the heart, and it is recommended for qi stagnation and blood stasis. TXLC is suitable for addressing the syndrome of deficiency of heart qi and obstruction of blood stasis collaterals, as it can replenish qi, activate blood, unblock the collaterals, and relieve pain. In summary, the six CTPMs have the effect of replenishing qi and activating blood, all of them appear to target the pathogenesis of HFpEF, and all exhibit positive therapeutic effects in NMA. However, the six CTPMs displayed different therapeutic advantages, which may be due to the fact that although all of them can boost qi and invigorate blood, their effects are focused differently. QLQXC can also warm yang and promote urination and is mostly used for patients who have developed edema due to yang deficiency in the late stage of the disease, rendering it more suitable for the treatment of HFrEF (92). YXSC focuses on replenishing qi and nourishing yin and is suitable for those with deficiency of both qi and yin, but the role of yin deficiency in the pathogenesis of HFPEF is less important. TXLC is a representative drug for the treatment of cardiovascular diseases guided by the theory of collateral disease, with strong blood-activating effects but relatively weak beneficial effects on qi. YXST has a stronger tonic effect and a weaker blood activating effect. SXBXP also regulates qi, which can strengthen the effect of activating blood, thus showing a better therapeutic effect. In comparison, QSYQDP includes Astragali Radix and Salviae Miltiorrhizae Radix et Rhizoma as the main active ingredients; they are strong in benefiting qi and invigorating blood, which may be the reason for the best efficacy of this treatment. The results of this study show that although all CTPMs can replenish qi and promote blood circulation, they produce different therapeutic effects due to their differences in functional focus. Hence, the use of a CTPM that specifically targets the syndromes of a disease can further improve clinical efficacy.

The complex pathogenesis of HFpEF may include left ventricular diastolic dysfunction, blood volume overload, microvascular dysfunction, microvascular inflammation, oxidative stress, myocardial metabolic dysfunction, abnormal myocardial cell structure, glucolipid metabolism, and subclinical systolic dysfunction (93–95). Experimental studies have shown that the active components of QSYQDP have good effects on improving ventricular diastolic function, antioxidative stress, anti-inflammation, anti-atherosclerosis and modulation of glucolipid metabolism (96, 97). SXBXP has pharmacological effects such as dilating coronary arteries, improving microcirculation, improving vascular endothelial function, inhibiting vascular wall inflammation and promoting therapeutic angiogenesis (98). The fact that QSYQDP and SXBXP act on key aspects of the pathomechanism of HFPEF is the basis for their better clinical efficacy.

### Innovations and limitations of the study

This study has a certain exploratory nature. The results showed integrative treatment to be superior to CWM treatment alone for HFpEF. The comprehensive efficacies of QSYQDP + CWM and SXBXP + CWM were most prominent. Among the six CTPMs included, only the package insert of QLQXC clearly specified its applicability to patients with HF; the package inserts of the other five CTPMs did not clearly mention their therapeutic effects on HF. However, a number of clinical studies have reported their therapeutic effects on HFpEF, indicating the possibility of applying medications beyond the uses indicated in package inserts. Treatment based on syndrome differentiation is a principle of TCM: the same treatments for the same syndromes and different treatments for different syndromes. Clinical medication should be applied primarily based on syndromes, with consideration of the specific diseases. In general, precise treatment based on syndrome differentiation is essential, though clinical application of a medicine is not necessarily restricted to the uses indicated in the package insert. This study analyzed existing evidence on both evidence-based medicine and syndrome differentiation-based TCM treatment to explore the possibility that some CTPMs can treat HFpEF, thus providing a refence for expanding the application of existing CTPMs, such as QSYQDP and SXBXP, and for the research and development of new CTPMs.

This study still has certain limitations. (1) Due to the limited published evidence, this study did not address the long-term prognosis of HFpEF patients, such as the mortality rate or the incidence of major adverse cardiovascular events. (2) The included RCTs were all from China, perhaps because the use of CTPMs is mainly limited to China. (3) The overall quality of the included RCTs was not high due to unclear and inadequate randomization and allocation concealment, failure to implement blinding, and potentially unstandardized baselines. (4) The small number of included RCTs and the small sample sizes for some interventions may have resulted in false-positive results in some comparisons or rankings, as well as the possibility of unpublished negative results. (5) The included RCTs involved complex potential causes of HFpEF, different disease durations, different types of CWM, and different lengths of intervention, thus reducing comparability between them and increasing clinical heterogeneity. In addition, the impact of these covariates on outcome indicators was not quantitatively evaluated through meta-regression analysis. (6) The clinical heterogeneity of relevant safety reports was high, and different measurement methods were used in different RCTs; therefore, quantitative pooled evaluation could not be performed.

## Conclusion

Available clinical evidence shows that compared with CWM alone, CTPMs + CWM combinations are superior at improving NYHA cardiac functional classification efficiency, E/A, NT-proBNP, and 6MWT and are safe for patients with HFpEF. Among the included interventions, QSYQDP + CWM and SXBXP + CWM may be the potential optimal treatments for HFpEF. Given the limitations of this study, further high-quality, multicenter, large sample, randomized, and double-blind studies are needed to confirm the current results.

## Data Availability

The original contributions presented in the study are included in the article/supplementary materials, further inquiries can be directed to the corresponding author.
