# Supplementary material for: Efficacy of Chinese traditional patent medicines for heart failure with preserved ejection fraction: a Bayesian network meta-analysis of 64 randomized controlled trials
Source: Front Cardiovasc Med. 2023 Nov 20;10:1255940. doi: 10.3389/fcvm.2023.1255940 (PMC10694238; doi:10.3389/fcvm.2023.1255940)
Supplement: Supplementary file 2 [file Table2.docx]

**Supplementary material S2 Criteria for judging risk of bias**

| Items | Risk | Judgment criteria |
| --- | --- | --- |
| Random sequence | low | The investigators describe a random component in the sequence generation process such as:  • referring to a random number table;  • using a computer random number generator;  • coin tossing;  • shuffling cards or envelopes;  • throwing dice;  • drawing of lots;  • minimization.*  *Minimization may be implemented without a random element, and this is considered to be equivalent to being random. |
|  | high | The investigators describe a non-random component in the sequence generation process. Usually, the description would involve some systematic, non-random approach, for example:  • sequence generated by odd or even date of birth;  • sequence generated by some rule based on date (or day) of admission;  • sequence generated by some rule based on hospital or clinic record number.  Other non-random approaches happen much less frequently than the systematic approaches mentioned here and tend to be obvious. They usually involve judgement or some method of non-random categorization of participants, for example:  • allocation by judgement of the clinician;  • allocation by preference of the participant;  • allocation based on the results of a laboratory test or a series of tests;  • allocation by availability of the intervention. |
|  | unclear | Insufficient information about the sequence generation process available to permit a judgement of ‘low risk’ or ‘high risk’. |
| Allocation concealment | low | Participants and investigators enrolling participants could not foresee assignment because one of the following, or an equivalent method, was used to conceal allocation:  • central allocation (including telephone, web-based and pharmacy-controlled randomization);  • sequentially numbered drug containers of identical appearance;  • sequentially numbered, opaque, sealed envelopes. |
|  | high | Participants or investigators enrolling participants could possibly foresee assignments, and thus introduce selection bias, due to allocation based on:  • use of an open random allocation schedule (e.g. a list of random numbers);  • use of assignment envelopes without appropriate safeguards (e.g. if envelopes were unsealed or nonopaque or not sequentially numbered);  • alternation or rotation;  • date of birth;  • case record number;  • any other explicitly unconcealed procedure. |
|  | unclear | Insufficient information available to permit a judgement of ‘low risk’ or ‘high risk’. This is usually the case if the method of concealment is not described or not described in sufficient detail to allow a definite judgement – for example if the use of assignment envelopes was described, but it remains unclear whether envelopes were sequentially numbered, opaque and sealed. |
| Blinding of patients and researchers | low | Either of the following:  • no blinding or incomplete blinding, but the review authors judge that the outcome was not likely to be influenced by lack of blinding;  • blinding of participants and key study personnel ensured, and unlikely that the blinding could have been broken. |
|  | high | Either of the following:  • no blinding or incomplete blinding, and the outcome was likely to be influenced by lack of blinding;  • blinding of key study participants and personnel attempted, but likely that the blinding could have been broken, and the outcome was likely to be influenced by lack of blinding. |
|  | unclear | Either of the following:  • insufficient information available to permit a judgement of ‘low risk’ or ‘high risk’;  • the study did not address this outcome. |
| Blinding of outcome evaluators | low | Either of the following:  • no blinding of outcome assessment, but the review authors judge that the outcome measurement was not likely to be influenced by lack of blinding;  • blinding of outcome assessment ensured, and unlikely that the blinding could have been broken. |
|  | high | Either of the following:  • no blinding of outcome assessment, and the outcome measurement was likely to be influenced by lack of blinding;  • blinding of outcome assessment, but likely that the blinding could have been broken, and the outcome measurement was likely to be influenced by lack of blinding. |
|  | unclear | Either of the following:  • insufficient information available to permit a judgement of ‘low risk’ or ‘high risk’;  • the study did not address this outcome. |
| Integrity of outcome data | low | Any one of the following:  • no missing outcome data;  • reasons for missing outcome data unlikely to be related to true outcome (for survival data, censoring unlikely to be introducing bias);  • missing outcome data balanced in numbers across intervention groups, with similar reasons for missing data across groups;  • for dichotomous outcome data, the proportion of missing outcomes compared with the observed event risk is not enough to have had a clinically relevant impact on the intervention effect estimate;  • for continuous outcome data, plausible effect size (difference in means or standardized difference in means) among missing outcomes is not enough to have had a clinically relevant impact on the observed effect size;  • missing data have been imputed using appropriate methods. |
|  | high | Any one of the following:  • reason for missing outcome data is likely to be related to true outcome, with either imbalance in numbers or reasons for missing data across intervention groups;  • for dichotomous outcome data, the proportion of missing outcomes compared with the observed event risk is enough to have induced clinically relevant bias in the intervention effect estimate;  • for continuous outcome data, plausible effect size (difference in means or standardized difference in means) among missing outcomes is enough to have induced clinically relevant bias in the observed effect size;  • ‘as-treated’ analysis done with substantial departure of the intervention received from that assigned at randomization;  • potentially inappropriate application of simple imputation. |
|  | unclear | Either of the following:  • insufficient reporting of attrition/exclusions to permit a judgement of ‘low risk’ or ‘high risk’ (e.g. number randomized not stated, no reasons for missing data provided);  • the study did not address this outcome. |
| Selective reporting of results | low | Either of the following:  • the study protocol is available and all of the study’s prespecified (primary and secondary) outcomes that are of interest in the review have been reported in the prespecified way;  • the study protocol is not available but it is clear that the published reports include all expected outcomes, including those that were prespecified (convincing text of this nature may be uncommon). |
|  | high | Any one of the following:  • not all of the study’s prespecified primary outcomes have been reported;  • one or more primary outcomes have been reported using measurements, analysis methods or subsets of the data (e.g. subscales) that were not prespecified;  • one or more reported primary outcomes were not prespecified (unless clear justification for their reporting is provided, such as an unexpected adverse effect);  • one or more outcomes of interest in the review have been reported incompletely so that they cannot be entered in a meta-analysis;  • the study report failed to include results for a key outcome that would be expected to have been reported for such a study. |
|  | unclear | Insufficient information available to permit a judgement of ‘low risk’ or ‘high risk’. It is likely that the majority of studies will fall into this category. |
| Other sources of bias | low | The study appears to be free of other sources of bias. |
|  | high | There is at least one important risk of bias. For example, the study:  • had a potential source of bias related to the specific study design used;  • has been claimed to have been fraudulent;  • had some other problem. |
|  | unclear | There may be a risk of bias, but there is either:  • insufficient information to assess whether an important risk of bias exists;  • insufficient rationale or evidence that an identified problem will introduce bias. |

Higgins JPT, Thomas J, Chandler J, Cumpston M, Li T, Page MJ, Welch VA. (2017). Cochrane Handbook for Systematic Reviews of Interventions (version 5.2). Cochrane. Available from www.training.cochrane.org/handbook.
