# Supplementary material for: Efficacy of Chinese traditional patent medicines for heart failure with preserved ejection fraction: a Bayesian network meta-analysis of 64 randomized controlled trials
Source: Front Cardiovasc Med. 2023 Nov 20;10:1255940. doi: 10.3389/fcvm.2023.1255940 (PMC10694238; doi:10.3389/fcvm.2023.1255940)
Supplement: Supplementary file 3 [file Table3.docx]

**Supplementary material S3**

**Source, compositions, amount of each composition, extraction procedure, actions, indications, administrations and chemical analysis of each Chinese Traditional Patent Medicine (CTPM)**

| **CTPMs** | **Source** | **compositions** | **Amount of each composition** | **Processing of each composition** | **Extraction procedure**  **of each CTPM** | **Actions of each CTPM** | **Indications** **of each CTPM** | **Administration and dosage** **of each CTPM** | **Quality control reported? (Y/N)** **of each CTPM** | **Chemical analysis  reported? (Y/N)** **of each CTPM** |
| --- | --- | --- | --- | --- | --- | --- | --- | --- | --- | --- |
| Qili Qiangxin Capsule | Shijiazhuang Yiling Pharmaceutical Co., Ltd. | *Astragalus membranaceus* (Fisch) Bge. Var. *mongholicus* (Bge.) Hsiao. [Leguminosae, Astragali Radix] | 450 g | Eliminate foreign matter, grade according to size, wash clean, soften thoroughly, cut into thick slices and dry. | Heat under reflux Astragals Radix, Descurainiae semen, Alismatis Rhizom, Ginseng Radix et Rhizoma and Periplocae Cortex with 70% ethanol twice, 3 hours for the first time and 2 hours for the second time, filter, combine the filtrates, concentrate to a thick extract with a relative density of 1.25-1.30 (60℃), and keep for later use. Distill Cinnamomi Ramulus and Citri Pericarpium Retculatae with water, collect the volatile oil, filter the aqueous solution, keep the filtrate for later use; decoct the drug residue with water for l hour, filter, combine the filtrate with the above filtrate, and keep for later use. Decoct Aconiti Lateralis Radix Praeparata, Salviae Miltiorrhizae Rhizoma et Radix; Polygonati odorati Rhizoma and Cartharni Flos with water twice, two hours for each time, combine the decoctions, filter, concentrate the filtrate to a thick extract with a relative density of l. 25-1. 30 (60℃), add ethanol to a content of 70% of ethanol in the extract, allow to stand for 24 hours at 4℃, filter, recover ethanol of the filtrate in vacuum, concentrate to a thick extract with a relative density of 1. 25-1. 30 (60℃), combine with the above thick extract, dry at 60-70℃. Pulverize the dried extract into fine Powder add a quantity of dextrin, make granules, spray the volatile oil, mix well and pack 1000 capsules. | To tonify qi, warm yang, activate blood, unblock the collaterals, promote urination and alleviate edema | Mild and moderate congestive heart failure of yang qi deficiency, obstruction of collaterals and water retention manifested as palpitations and shortness of breath worsening upon physical movement, difficulty in lying down, edema in the lower limbs, fatigue, lack of strength, scanty urine, cyanotic mouth and lips, fear of cold with cold limbs. and expectoration of thin white sputum; Coronary heart disease and hypertension with the symptoms described above. | 0. 3 g per capsule.  For oral administration, 4 capsules, 3 times a day. | Y - ZYB2072011007 issued by National Medical Preducts Administration [Detail information can be got from https://www.nmpa.gov.cn/directory/web/nmpa/xxgk/ggtg/zhybhpzh/zhybhpzhgg/20110626120001140.html] | Y - HPLC [Detail information can be got from Pharmacopoeia of the People's Republic of China (Part I, finished preparations and single flavor preparations, Qili Qiangxin Jiaonang)] |
|  |  | *Panax ginseng* C. A. Mey. [Araliaceae, Ginseng Radix et Rhizoma] | 225 g | Soften thoroughly, cut into thin slices, and dry, or pulverize or break to pieces before use. |  |  |  |  |  |  |
|  |  | *Aconitum carmichaeli* Debx. [Ranunculaceae, Aconiti Lateralis Radix Praeparata] | 112.5 g | Fupian “Heishunpain” and “Baifupian” are used directly. |  |  |  |  |  |  |
|  |  | *Salvia miltiorrhiza* Bge. [Lamiaceae, Salviae Miltiorrhizae Radix et Rhizoma] | 225 g | Remove impurities and residual stems, wash, moisten thoroughly, cut thick slices and dry. |  |  |  |  |  |  |
|  |  | *Descurainia Sophia* (L.) Webb ex Prantl or *Lepidium apetalum* Willd. [Cruciferae, Descurainiae Semen Lepidii Semen] | 150 g | Descurainiae Semen or Lepidii Semen Eliminate foreign mailer. |  |  |  |  |  |  |
|  |  | *Alisma orientate* (Sam) Juzep. [Alismataceae, Alismatis Rhizoma] | 225 g | Alismatis Rhizoma Eliminate foreign matter, soak slightly, soften thoroughly, cut into thick slices, and dry. |  |  |  |  |  |  |
|  |  | *Polygonatum odoratum* (Mill.) Druce. [Liliaceae, Polygonati Odorati Rhizoma] | 75 g | Eliminate foreign matter, wash clean, soften thoroughly, cut into thick slices or sections and dry. |  |  |  |  |  |  |
|  |  | *Cinnamomum cassia* (L.) J.Presl. [Lauraceae, Cinnamomi Ramulus] | 90 g | Remove impurities, wash, moisten thoroughly, cut into thick pieces and dry. |  |  |  |  |  |  |
|  |  | *Carthamus tinctorius* L. [Compositae, Carthami Flos] | 90 g | Remove impurities. |  |  |  |  |  |  |
|  |  | *Periploca sepium* Bge. [Asclepiadaceae, Periplocae Cortex] | 180 g | Eliminate foreign matter, wash clean, soften thoroughly, cut into thick slices, and dry in the sun. |  |  |  |  |  |  |
|  |  | *Citrus reticulata Blanco* or its cultivars [Rutaceae, Citri Reticulatae Pericarpium] | 75 g | Eliminated foreign maller, spray with. water, soften thoroughly, cut into slivers, dry in the shade. |  |  |  |  |  |  |
| Qishen Yiqi dropping pill | Tianjin Tianshili Pharmaceutical Co., Ltd. | *Astragalus membranaceus* (Fisch) Bge. Var. *mongholicus* (Bge.) Hsiao. [Leguminosae, Astragali Radix] | 900 g | Eliminate foreign matter, grade according to size, wash clean, soften thoroughly, cut into thick slices and dry. | The above four ingredients, Salviae Miltiorrhizae Radix et Rhizoma and Notoginseng Radix et Rhizoma were decocted twice with water for 2 hours each time, filtered, and the filtrate was concentrated to a relative density of 1. 13-1. 23(80°C), ethanol was added to reach 70% alcohol content, allowed to stand, filtered, and the filtrate was recovered from ethanol and concentrated into a thick paste; Astragali Radix was decocted twice with water for 2 hours the first time and 1 hour the second time, filtered, and the filtrate was concentrated to a relative density of 1. 05-1. 20(75°C). The filtrate was concentrated to a relative density of 1. 18~1. 30(60°C), ethanol was added to bring the alcohol content to so%, left to stand, filtered, the filtrate was recovered and concentrated into a thick paste. Combine the above two thick paste, add the most suitable polyethylene glycol 6000, heat and melt, add sorbitol oil, mix well, and make 1050g of dropping pills, or wrap the film coating, that is. | To tonify qi, free the vessels, active blood and stop pain | Chest *bi* disorder caused by qi deficiency and blood stasis, symptoms include chest tightness and chest pain, shortness of breath and fatigue, palpitations, spontaneous sweating, pale complexion, fat tongue with teeth marks, dark tongue or paralysis spots, and deep and stringy pulse; Angina pectoris in coronary heart disease with the symptoms described above. | 0.5g per bag.  For oral administration, 1bag each time, 3 times a day. | Y - ZYB20720170060 issued by National Medical Preducts Administration [Detail information can be got from https://www.nmpa.gov.cn/directory/web/nmpa/xxgk/ggtg/zhybhpzh/zhybhpzhgg/20170725170901352.html] | Y -  HPLC  [Detail information can be got from Pharmacopoeia of the People's Republic of China (Part I, finished preparations and single flavor preparations, Qishen Yiqi Diwan)] |
|  |  | *Salvia miltiorrhiza* Bge. [Lamiaceae, Salviae Miltiorrhizae Radix et Rhizoma] | 900 g | Remove impurities and residual stems, wash, moisten thoroughly, cut thick slices and dry. |  |  |  |  |  |  |
|  |  | *Panax notoginseng* (Burkill) F.H.Chen [Araliaceae, Notoginseng Radix et Rhizoma] | 180 g | Take Panax notoginseng, wash, dry and grind into fine powder |  |  |  |  |  |  |
|  |  | *Dalbergia odorifera* T. Chen [Legumes, Dalbergiae Odoriferae Lignum] | 12 g | Extraction of volatile oil from Dalbergiae Odoriferae Lignum by refluxing with water |  |  |  |  |  |  |
| Yixinshu capsule | Guizhou Xinbang  Pharmaceutical Co., Ltd. | *Panax ginseng* C. A. Mey. [Araliaceae, Ginseng Radix et Rhizoma] | 200 g | Soften thoroughly, cut into thin slices, and dry, or pulverize or break to pieces before use. | The above seven ingredients, Pulverize Ginseng Radix et Rhizoma to fine powder; Extract Schisandrae Chinensis Fructus, Salviae Miltiorrhizae Radix et Rhizoma with 85% ethanol under reflux twice, 3 hours for the first time and 1.5 hours for the second time, filter, combine the filtrate, recover ethanol and concentrate in vaccum to form a thick extract with a relative density of 1. 25-1. 30 (80℃). Decoct other four ingredients with water for twice, 2. 5 hours for the first time and 1.5 hours for the second time, filter, combine the filtrate, concentrate in vaccum to form a thick extract with a relative density of 1. 10-l. 15 (80℃), add the same amount of 85% ethanol and mix well, allow to stand, filter, combine the filtrate, recover ethanol and concentrate to an appropriate quantity, mix well with the above extract of Schisandrae Chinensis Fructus and Salviae Miltiorrhizae Radix et Rhizoma, add a quantity of powder of Ginseng Radix et Rhizoma and appropriate quantity of starch, dry, Pulverize to fine powder, make to 1000 capsules. | To tonify qi, restore the pulse, activate blood, resolve stasis, nourish yin and engender fluid. | Chest *bi* disorder due to dual deficiency of qi and yin, or static blood obstructing collaterals, manifested as pain and oppression in the chest, palpitations, shortness of breath, intermittent pulses; Angina pectoris in coronary heart disease with the symptoms described above. | 0. 4 g per capsule.  For oral administration, 3  capsules per time, three times a day. | Y -  ZYB20799112 issued by National Medical Preducts Administration  [Detail information can be got from https://www.nmpa.gov.cn/xxgk/ggtg/zhybhpzh/zhybhpzhgg/19990510010101955.html] | Y -  HPLC  [Detail information can be got from Pharmacopoeia of the People's Republic of China (Part I, finished preparations and single flavor preparations, Yixinshu Jiaonang)] |
|  |  | *Ophiopogon japonicus* (Thunb.) Ker Gawl. [Asparagaceae, Ophiopogonis Radix] | 200 g | Remove impurities, wash, moisten, flatten and dry. |  |  |  |  |  |  |
|  |  | *Schisandra chinensis* (Turcz.) Barll. [Magnoliaceae, Schisandrae Chinensis Fructus] | 133 g | Eliminate foreign matter.  Break into pieces before use. |  |  |  |  |  |  |
|  |  | *Astragalus membranaceus* (Fisch) Bge. Var. *mongholicus* (Bge.) Hsiao. [Leguminosae, Astragali Radix] | 200 g | Eliminate foreign matter, grade according to size, wash clean, soften thoroughly, cut into thick slices and dry. |  |  |  |  |  |  |
|  |  | *Salvia miltiorrhiza* Bge. [Lamiaceae, Salviae Miltiorrhizae Radix et Rhizoma] | 267 g | Remove impurities and residual stems, wash, moisten thoroughly, cut thick slices and dry. |  |  |  |  |  |  |
|  |  | *Ligusticum striatum* DC. [Apiaceae, Chuanxiong Rhizoma] | 133 g | Remove impurities, separate size, wash, moisten thoroughly, cut thick pieces and dry |  |  |  |  |  |  |
|  |  | *Crataegus pinnatifida* Bunge [Rosaceae, Crataegi Fructus] | 200 g | Remove impurities and exfoliated nuclei |  |  |  |  |  |  |
| Yangxinshi tablet | Qingdao Guofeng  Pharmaceutical Co., Ltd. | *Astragalus membranaceus* (Fisch) Bge. Var. *mongholicus* (Bge.) Hsiao. [Leguminosae, Astragali Radix] | 120 g | Eliminate foreign matter, grade according to size, wash clean, soften thoroughly, cut into thick slices and dry. | Pulverize Ginseng Radix et Rhizoma, Coptidis Radix, Corydalis Radix (processed with vinegar), Crateagi Fructus and 60 g of Astragali Radix to fine powder. Dccoct the remaining Astragali Radix and other eight ingredients with water twice, 2 hours for the first time and 1. 5 hours for the second time, filter and combine the filtrates. Concentrate the filtrate to a thin extract with a relative density of 1. 06- 1. 12 (92℃), allow to cool, add 1. 5 times quantity of ethanol to precipitate, allow to stand, and filter. Concentrate the filtrate to a thin extract with a relative density of 1. 20- 1. 22 (90℃). Mix the concentrated extracts well with the above powder, make granules, dry, compress into 1000 tablets (small tablets), and coat with sugar or film, or compress into 500 tablets (big tablets) and coal with film. | To tonify qi, activate blood, resolve stasis and relieve pain. | Chest *bi* disorder due to qi deficiency with blood stasis, manifested as palpitations, shortness of breath, oppression in the chest, stabbing pain of precordium; angina pectoris in coronary heart disease with the symptoms described above. | 0. 3 g per film coated tablet or 0. 6 g per film coated tablet.  For oral administration, 4-6 tablets (0.3g/tablet) per time or 2-3 tablets (0.6g/tablet) per time, three times a day. | Y -  ZYB20720010540 issued by National Medical Preducts Administration  [Detail information can be got from https://www.nmpa.gov.cn/directory/web/nmpa/xxgk/ggtg/zhybhpzh/zhybhpzhgg/20001029010101865.html] | Y -  HPLC  [Detail information can be got from Pharmacopoeia of the People's Republic of China (Part I, finished preparations and single flavor preparations, Yangxinshi Pian)] |
|  |  | *Codonopsis pilosula* (Franch.) Nannf. or *Codonopsis tangshen* Oliv. [Campanulaceae, Codonopsis Radix] | 100 g | Eliminate foreign matter, wash clean, soften thoroughly, cut into thick slices, and dry. |  |  |  |  |  |  |
|  |  | *Salvia miltiorrhiza* Bge. [Lamiaceae, Salviae Miltiorrhizae Radix et Rhizoma] | 80 g | Remove impurities and residual stems, wash, moisten thoroughly, cut thick slices and dry. |  |  |  |  |  |  |
|  |  | *Pueraria montana* var. *lobata* (Willd.) Sanjappa & Pradeep [Leguminosae, Puerariae Lobatae Radix] | 80 g | Remove impurities, wash, moisten thoroughly, cut into thick pieces and dry in the sun |  |  |  |  |  |  |
|  |  | *Epimedium brevicornu* Maxim.or *Epimedium sagittatum* (Sieb. et Zucc.) Maxim. [Berberidaceae, Epimedii Folium] | 80 g | Eliminate foreign matter, pick the leaves, spray with water, soften slightly, cut into slivers and dry. |  |  |  |  |  |  |
|  |  | *Crataegus pinnatifida* Bunge [Rosaceae, Crataegi Fructus] | 80 g | Remove impurities and exfoliated nuclei. |  |  |  |  |  |  |
|  |  | *Rehjnannia glutinosa* Libosch. [Scrophulariaceae, Rehmanniae Radix] | 60 g | Eliminate foreign matter, wash clean, cover to soften, cut into thick slices , and dry. |  |  |  |  |  |  |
|  |  | *Angelica sinensis* (Oliv.) Diels [Apiaceae, Angelicae Sinensis Radix] | 60 g | Remove impurities, wash, moisten, slice, dry in the sun or at low temperature. |  |  |  |  |  |  |
|  |  | *Coptis chinensis* Franch., *Coptis deltoidea* C. Y. Cheng et Hsiao or *Coptis teeta* Wall. [Ranunculaceae, Coptidis Rhizoma] | 60 g | Eliminate foreign matter,  soften thoroughly, cut into thin slices, dry in air, or break to pieces before use. |  |  |  |  |  |  |
|  |  | *Corydalis yanhusuo* W. T. Wang. [Papaveraceae, Corydalis Rhizoma] | 60 g | Eliminate foreign matter, wash clean, dry, and cut into thick slices or break to pieces before use. |  |  |  |  |  |  |
|  |  | *Ganoderma lucidum* (Leyss. ex Fr.) Karst. [Polyporaceae, Ganoderma] | 60 g | Eliminate foreign matter, attach rotten wood, dry in the shade or stove at 40-50℃. |  |  |  |  |  |  |
|  |  | *Panax ginseng* C. A. Mey. [Araliaceae, Ginseng Radix et Rhizoma] | 25 g | Soften thoroughly, cut into thin slices, and dry, or pulverize or break to pieces before use. |  |  |  |  |  |  |
|  |  | *Glycyrrhiza uralensis* Fisch., *Glycyrrhiza inflata* Bat. or *Glycyrrhiza glabra* L. [Leguminosae, Glycyrrhizae Radix Et Rhizoma Praeparata Cum Melle] | 25 g | Stir-bake the slices of Glycyrrhizae Radix as  described under the method for stir-baking with honey (0213) until it becomes yellow to deep yellow and not sticky  to the fingers, take out and cool in the air. |  |  |  |  |  |  |
| Shexiang Baoxin Pill | Shanghai Hehuang  Pharmaceutical Co., Ltd. | *Moschus berezovskii* Flerov., *Moschus sifanicus* Przewalski or *Moschus moschiferus* Linnaeu. [Cervidae, Moschus Artifactus] | Unpublished | Remove the sac wall of “Maokeshexiang”, take out the “Shexiangren” and eliminate foreign matter'.  Pulverize before use. | Pulverize the six ingredients except Styrax to fine powder, make pills with Styrax and a quantity of white wine and dry. | To warm and unblock meridians with aromatic medicinals, replenish qi and strengthen heart. | Chest *bi* disorder due to qi stagnation and blood stasis, manifested as fixed pain in precardium; Angina pectoris and myocardial infarction due to myocardial ischemia with the symptoms described above. | 22. 5 mg per pill.  For oral administration, 1-2 pills per time, three times a day. | Y -  ZYB20794071 issued by National Medical Preducts Administration  [Detail information can be got from https://www.nmpa.gov.cn/xxgk/ggtg/zhybhpzh/zhybhpzhgg/19940206010101501.html] | Y -  HPLC  [Detail information can be got from Pharmacopoeia of the People's Republic of China (Part I, finished preparations and single flavor preparations, Shexiang Baoxin Wan)] |
|  |  | *Panax ginseng* C. A. Mey. [Araliaceae, Ginseng Radix et Rhizoma] | Unpublished | Soften thoroughly, cut into thin slices, and dry, or pulverize or break to pieces before use. |  |  |  |  |  |  |
|  |  | *Bos taurus domesticus* Gmelin. [Bovine, Bovis Calculus Artifactus] | Unpublished | Artificial Cow-bezoan is prepared with powder of cow bile, cholic acid, hyodeoxycholic acid, taurine, bilirubin, cholesterol and trace elements, etc. |  |  |  |  |  |  |
|  |  | *Cinnamomum cassia* Presl. [Lauraceae, Cinnamomi Cortex] | Unpublished | Eliminate foreign matter and rough bark. Pound to pieces before use. |  |  |  |  |  |  |
|  |  | *Liquidambar orientalis* Mill. [Hamamelidaceae, Styrax] | Unpublished | Purify Styrax to get purified balsam |  |  |  |  |  |  |
|  |  | *Bufo bufo gargarizans* Cantor *or Bufo melanostictus* Schneider (Bufonidae, Bufonis Venenum) | Unpublished | Break Bufonis Venenum to pieces, macerate with white rice wine, frequently stir until become concentrated extract, dry and pulverize. |  |  |  |  |  |  |
|  |  | *Cinnamomum camphora* (L.) Presl [Lauraceae, Borneolum] | Unpublished | Steam distillation. |  |  |  |  |  |  |
| Tongxinluo capsule | Shijiazhuang Yiling Pharmaceutical Co., Ltd | *Panax ginseng* C. A. Mey. [Araliaceae, Ginseng Radix et Rhizoma] | Unpublished | Soften thoroughly, cut into thin slices, and dry, or pulverize or break to pieces before use. | Make Capsules with the above ingredients. | To tonify qi, activate blood. unblock the collaterals and relieve pain. | Angina pectoris of coronary heart disease belongs to the syndrome of deficiency of heart qi and obstruction of blood stasis collaterals. The symptoms include suffocation, tingling and colic in the chest, immobility, palpitation, spontaneous sweating, shortness of breath and fatigue, purple dark tongue or ecchymosis, astringent pulse or generation. | 0.26 g per capsule.  For oral administration, 2-4 capsules per time, three times a day. | Y - ZYB20799098 issued by National Medical Preducts Administration [Detail information can be got from https://www.nmpa.gov.cn/xxgk/ggtg/zhybhpzh/zhybhpzhgg/19990510010101955.html] | Y - HPLC-Detail information can be got from Pharmacopoeia of the People's Republic of China (Part I, finished preparations and single flavor preparations, Tongxinluo Jiaonang) |
|  |  | *Hirudo nipponica* Whitman. [Haemadipsidae, Hirudo] | Unpublished | Wash, cut and dry |  |  |  |  |  |  |
|  |  | *Buthus martensii* Karsch. [Scorpionidae, Scorpio] | Unpublished | Remove impurities, wash and dry |  |  |  |  |  |  |
|  |  | *Paeonia lactiflora* Pall. [Paeoniaceae, Paeoniae Radix Rubra] | Unpublished | Remove impurities, separate size, wash, moisten thoroughly, cut thick pieces and dry |  |  |  |  |  |  |
|  |  | *Cryptotympana pustulata* Fabricius. [Cicadellidae, Cicadae Periostracum] | Unpublished | Remove impurities, wash and dry |  |  |  |  |  |  |
|  |  | *Eupolyphaga sinensis* Walker. [Blaberidae, Eupolyphaga Steleophaga] | Unpublished | Scald in boiling water, dry or dry |  |  |  |  |  |  |
|  |  | *Scolopendra subspinipes mutilans* L. Koch. [Scolopendridae, Scolopendra] | Unpublished | Remove bamboo slices, wash, bake yellow over low heat, and cut into sections |  |  |  |  |  |  |
|  |  | *Santalum album* L. [Sandalwood, Santali Albi Lignum] | Unpublished | Saw into sections about 3 cm long, then split to pieces or pulverize to coarse powder. |  |  |  |  |  |  |
|  |  | *Dalbergia odorifera* T. Chen [Legumes, Dalbergiae Odoriferae Lignum] | Unpublished | Extraction of volatile oil from Dalbergiae Odoriferae Lignum by refluxing with water |  |  |  |  |  |  |
|  |  | *Boswellia carterii* Birdw. or Boswellia hhaw-dajiana Birdw. [Burseraceae, Olibanum] | Unpublished | Take the pure frankincense and fry it with vinegar (general rule 0213) until the surface is bright.5kg vinegar per 100kg Boswellia sacra Flueck. |  |  |  |  |  |  |
|  |  | *Ziziphus jujuba* Mill. var. *spinosa (Bunge)* Hu ex H. F. Chou. [Rhamnaceae, Ziziphi Spinosae Semen] | Unpublished | Remove the remained  shells. Break to pieces before use. |  |  |  |  |  |  |
|  |  | *Cinnamomum camphora* (L.) Presl [Lauraceae, Borneolum] | Unpublished | Fresh branches and leaves are extracted and processed |  |  |  |  |  |  |

**Reference:**

Committee of National Pharmacopoeia. (2015). *Pharmacopoeia of the People's Republic of China*. Chemical Industry Press.

Committee of National Pharmacopoeia. (2020). *Pharmacopoeia of the People's Republic of China*. Chemical Industry Press.

Li, X., Zhang, J., Huang, J., Ma, A., Yang, J., Li, W., Wu, Z., Yao, C., Zhang, Y., Yao, W., Zhang, B., Gao, R., & Efficacy and Safety of Qili Qiangxin Capsules for Chronic Heart Failure Study Group (2013). A multicenter, randomized, double-blind, parallel-group, placebo-controlled study of the effects of qili qiangxin capsules in patients with chronic heart failure. Journal of the American College of Cardiology, 62(12), 1065–1072. <https://doi.org/10.1016/j.jacc.2013.05.035>

Wang, M., Shan, Y., Wu, C., Cao, P., Sun, W., Han, J., Shen, L., Chen, J., Yu, P., & Chen, X. (2021). Efficacy and Safety of Qishen Yiqi Dripping Pill for Heart Failure With Preserved Ejection Fraction: A Systematic Review and Meta-Analysis. Frontiers in pharmacology, 11, 626375. <https://doi.org/10.3389/fphar.2020.626375>

Han, J. Y., Li, Q., Pan, C. S., Sun, K., & Fan, J. Y. (2019). Effects and mechanisms of QiShenYiQi pills and major ingredients on myocardial microcirculatory disturbance, cardiac injury and fibrosis induced by ischemia-reperfusion. Pharmacological research, 147, 104386. <https://doi.org/10.1016/j.phrs.2019.104386>

Wang, M., Shan, Y., Sun, W., Han, J., Tong, H., Fan, M., Chen, J., Yu, P., Shen, L., & Chen, X. (2021). Effects of Shexiang Baoxin Pill for Coronary Microvascular Function: A Systematic Review and Meta-Analysis. Frontiers in pharmacology, 12, 751050. https://doi.org/10.3389/fphar.2021.751050

Li, J., Zhao, X., Zhang, Y., Wan, H., He, Y., Li, X., Yu, L., & Jin, W. (2021). Comparison of Traditional Chinese Medicine in the Long-Term Secondary Prevention for Patients with Ischemic Stroke: A Systematical Analysis. Frontiers in pharmacology, 12, 722975. <https://doi.org/10.3389/fphar.2021.722975>
