# Supplementary material for: Efficacy of Chinese traditional patent medicines for heart failure with preserved ejection fraction: a Bayesian network meta-analysis of 64 randomized controlled trials
Source: Front Cardiovasc Med. 2023 Nov 20;10:1255940. doi: 10.3389/fcvm.2023.1255940 (PMC10694238; doi:10.3389/fcvm.2023.1255940)
Supplement: Supplementary file 4 [file Table4.docx]

**Supplementary material S4 Risk of bias assessment results for each study**

| References | Random sequence generation (selection bias) | Allocation concealment (selection bias) | Blinding of participants and personnel (performance bias) | Blinding of outcome assessment (detection bias) | Incomplete outcome date (attrition bias) | Selective reporting (reporting bias) | other biases |
| --- | --- | --- | --- | --- | --- | --- | --- |
| Bu and Zhang, 2021 (18) | low | high | high | high | low | low | unclear |
| Chen et al., 2021 (19) | high | high | high | high | low | low | unclear |
| Chen et al., 2021 (20) | high | high | high | high | low | low | unclear |
| Chen, 2014 (21) | unclear | high | high | high | low | low | unclear |
| Cheng et al., 2019 (22) | unclear | high | high | high | low | low | unclear |
| Chi et al., 2014 (23) | unclear | high | high | high | low | low | unclear |
| Dai, 2015 (24) | low | high | high | high | low | low | unclear |
| Dong and Wang, 2012 (25) | unclear | high | low | high | low | low | unclear |
| Dong et al., 2018 (26) | unclear | high | high | high | low | low | unclear |
| Du, 2014 (27) | unclear | high | high | high | low | low | unclear |
| Du, 2022 (28) | low | high | high | high | low | low | unclear |
| Feng et al., 2014 (29) | unclear | high | high | high | low | low | unclear |
| Guan and Yang, 2012 (30) | unclear | high | high | high | low | low | unclear |
| He, 2015 (31) | unclear | high | high | high | low | low | unclear |
| Hou and Yang, 2019 (32) | low | high | high | high | low | low | unclear |
| Hu et al., 2015 (33) | unclear | high | high | high | low | low | unclear |
| Hu, et al., 2016 (34) | low | high | high | high | low | low | unclear |
| Huang et al., 2009 (35) | low | high | high | high | low | low | unclear |
| Huang et al., 2017 (36) | low | high | low | low | low | low | unclear |
| Jian and Ji, 2012 (37) | unclear | high | high | high | low | low | unclear |
| Jian et al., 2015 (38) | high | high | high | high | low | low | unclear |
| Li and Liu, 2015 (39) | high | high | high | high | low | low | unclear |
| Li et al., 2012 (40) | unclear | high | high | high | low | low | unclear |
| Li et al., 2017 (41) | unclear | high | high | high | low | low | unclear |
| Li et al., 2018 (42) | high | high | high | high | low | low | unclear |
| Li, 2014 (43) | unclear | high | high | high | low | low | unclear |
| Li, 2015 (44) | unclear | high | high | high | low | low | unclear |
| Li, 2018 (45) | low | high | high | high | low | low | unclear |
| Liao, 2013 (46) | low | low | high | high | low | low | unclear |
| Liu et al., 2011 (47) | low | high | high | high | low | low | unclear |
| Liu et al., 2023 (48) | low | high | low | low | low | low | unclear |
| Liu, 2017 (49) | low | high | high | high | low | low | unclear |
| Liu, 2020 (50) | low | high | high | high | low | low | unclear |
| Liu, 2021 (51) | low | high | high | high | low | low | unclear |
| Lu et al., 2013 (52) | unclear | high | high | high | low | low | unclear |
| Luo and Wan, 2016 (53) | unclear | high | high | high | low | low | unclear |
| Ma et al., 2011 (54) | unclear | high | high | high | low | low | unclear |
| Pan, 2014 (55) | high | high | high | high | low | low | unclear |
| Pei et al., 2016 (56) | low | high | high | high | low | low | unclear |
| Peng et al., 2012 (57) | unclear | high | high | high | low | low | unclear |
| Qiang, 2016 (58) | unclear | high | high | high | low | low | unclear |
| Qiu, 2011 (59) | unclear | high | high | high | low | low | unclear |
| Ruan, 2021 (60) | low | high | high | high | low | low | unclear |
| Song, 2020 (61) | low | high | high | high | low | low | unclear |
| Sun, 2013 (62) | unclear | high | high | high | low | low | unclear |
| Tian, 2011 (63) | low | high | high | high | low | low | unclear |
| Wang et al., 2016 (64) | unclear | high | high | high | low | low | unclear |
| Wang, 2022 (65) | low | high | high | high | low | low | unclear |
| Wei, 2008 (66) | unclear | high | high | high | low | low | unclear |
| Wu et al., 2023 (67) | unclear | high | high | high | low | low | unclear |
| Xu, 2007 (68) | unclear | high | high | high | low | low | unclear |
| Yang et al., 2010 (69) | unclear | high | high | high | low | low | unclear |
| Yang et al., 2018 (70) | low | high | high | high | low | low | unclear |
| Yu, 2008 (71) | unclear | high | low | low | low | low | unclear |
| Yu, 2016 (72) | unclear | high | high | high | low | low | unclear |
| Yuan, 2012 (73) | unclear | high | high | high | low | low | unclear |
| Zeng and Zhu, 2004 (74) | high | high | high | high | low | low | unclear |
| Zhang and Gou, 2021 (75) | low | high | high | high | low | low | unclear |
| Zhang and Li, 2013 (76) | unclear | high | high | high | low | low | unclear |
| Zhang and Niu, 2017 (77) | low | high | high | high | low | low | unclear |
| Zhang and Zhu, 2011 (78) | low | high | high | high | low | low | unclear |
| Zhang et al., 2019 (79) | low | high | high | high | low | low | unclear |
| Zhang et al., 2021 (80) | unclear | high | high | high | low | low | unclear |
| Zhen and Tian, 2013 (81) | unclear | high | high | high | low | low | unclear |
