# Supplementary material for: Efficacy of Chinese traditional patent medicines for heart failure with preserved ejection fraction: a Bayesian network meta-analysis of 64 randomized controlled trials
Source: Front Cardiovasc Med. 2023 Nov 20;10:1255940. doi: 10.3389/fcvm.2023.1255940 (PMC10694238; doi:10.3389/fcvm.2023.1255940)
Supplement: Supplementary file 5 [file Table5.docx]

**Supplementary material S5 Model convergence for E/A, NT-proBNP, and 6MWT**


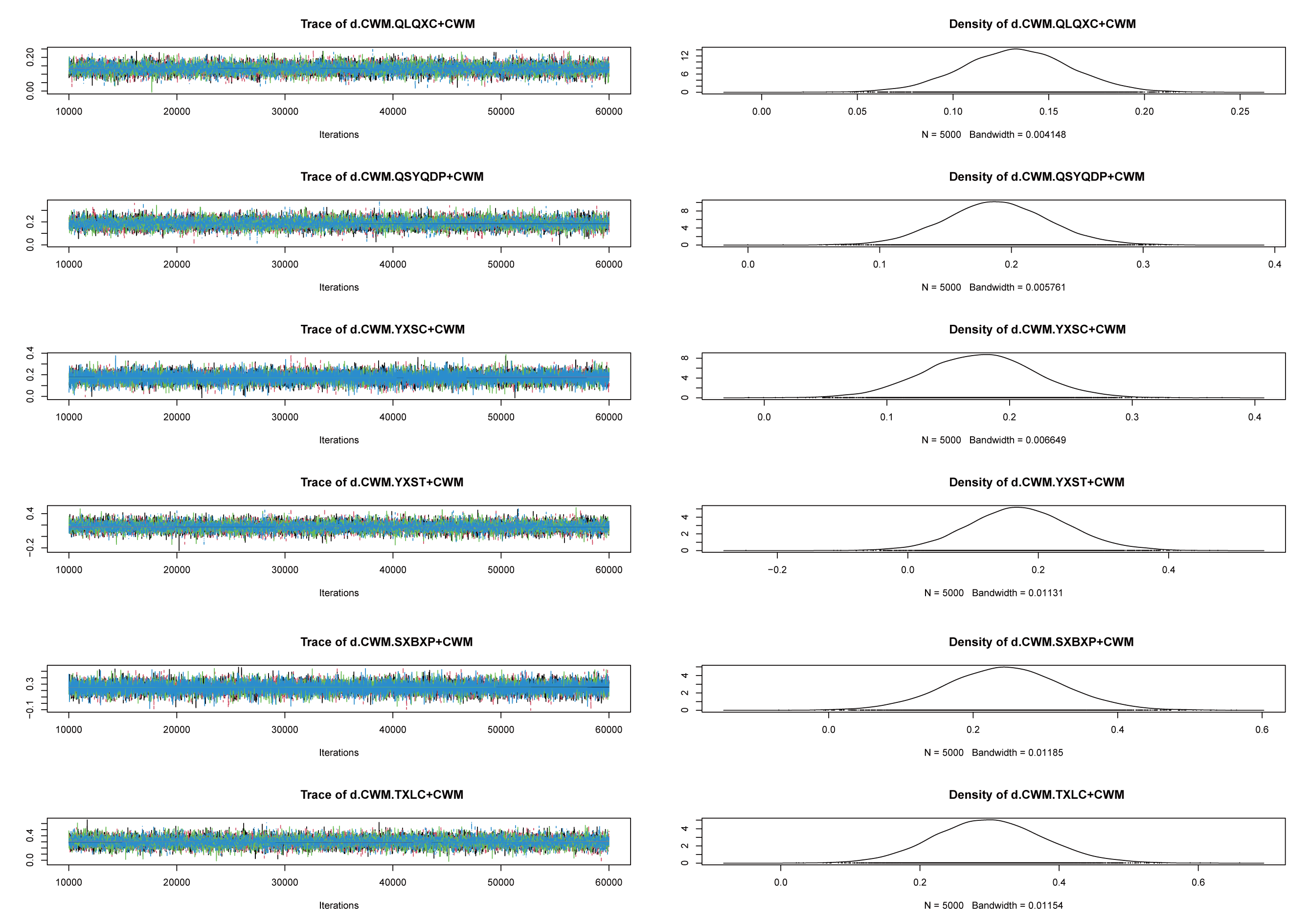


Trace plot and density plot for the ratio of early diastolic mitral inflow velocity to late diastolic mitral inflow velocity (E/A). CWM, conventional western medicine; QLQXC, Qili Qiangxin Capsule; QSYQDP, Qishen Yiqi dropping pill; YXSC, Yixinshu capsule; YXST Yangxinshi tablet; SXBXP, Shexiang Baoxin Pill; TXLC, Tongxinluo Capsule.


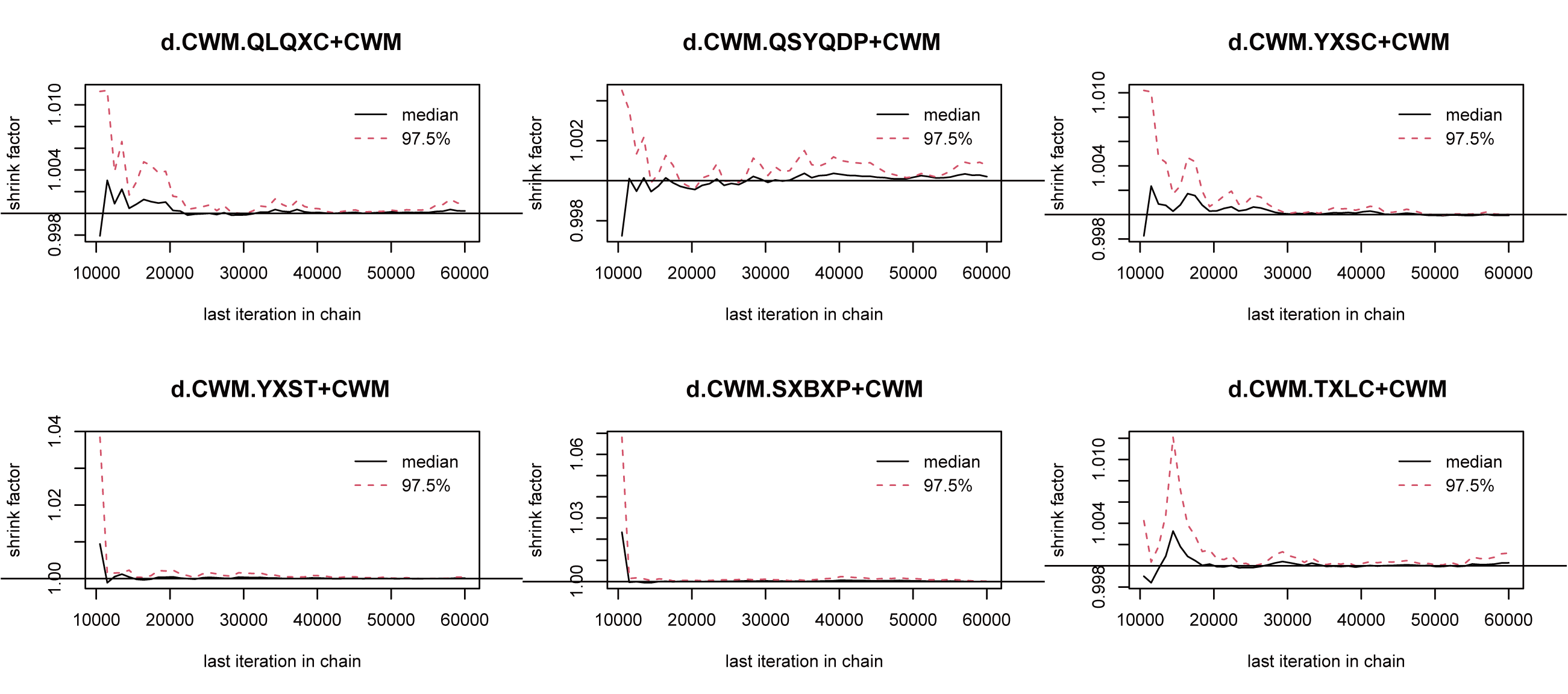


Brooks-Gelman-Rubin diagnosis plot for the ratio of early diastolic mitral inflow velocity to late diastolic mitral inflow velocity (E/A). CWM, conventional western medicine; QLQXC, Qili Qiangxin Capsule; QSYQDP, Qishen Yiqi dropping pill; YXSC, Yixinshu capsule; YXST Yangxinshi tablet; SXBXP, Shexiang Baoxin Pill; TXLC, Tongxinluo Capsule.


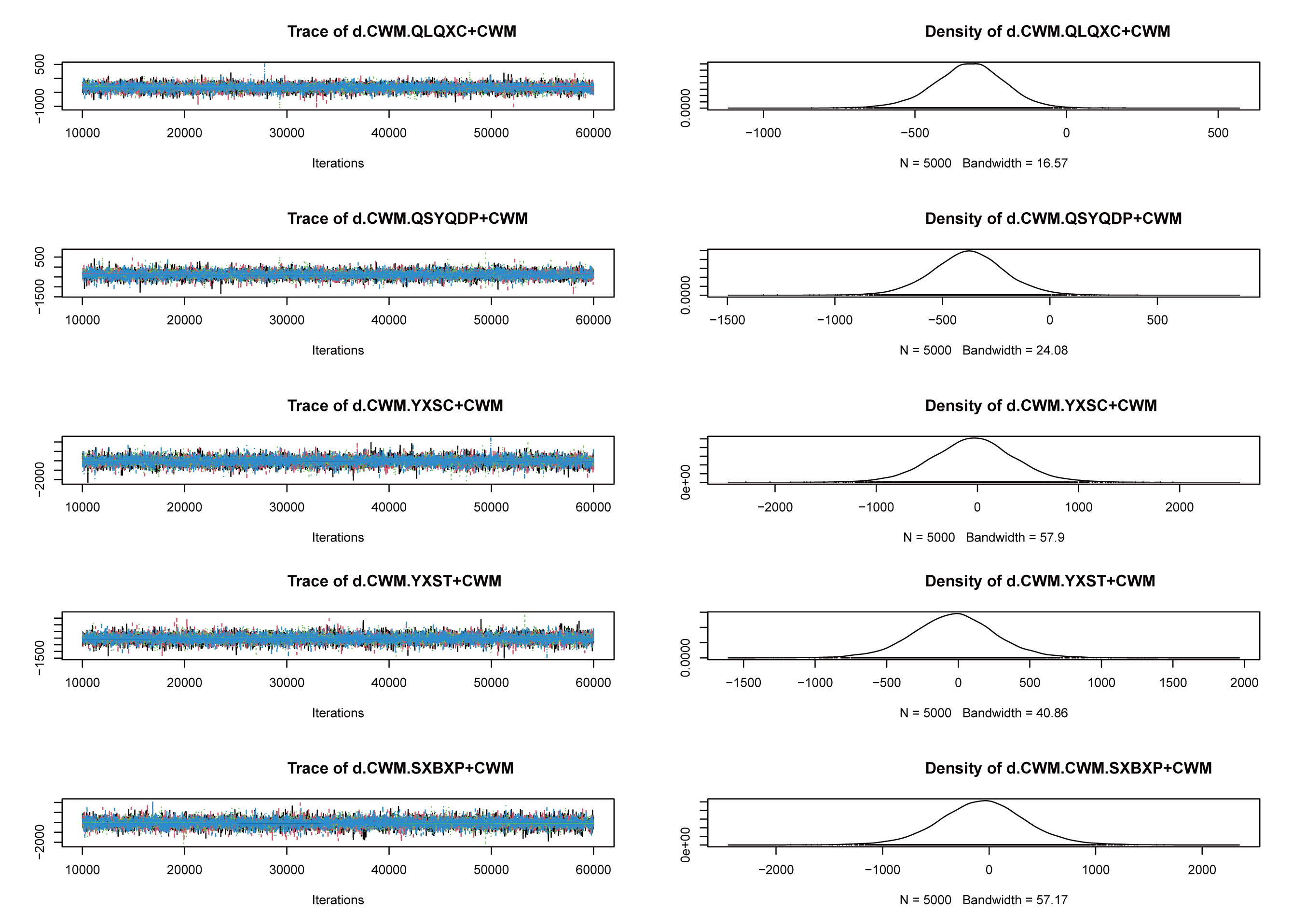


Trace plot and density plot for N-terminal pro-B type natriuretic peptide (NT-proBNP). CWM, conventional western medicine; QLQXC, Qili Qiangxin Capsule; QSYQDP, Qishen Yiqi dropping pill; YXSC, Yixinshu capsule; YXST Yangxinshi tablet; SXBXP, Shexiang Baoxin Pill.


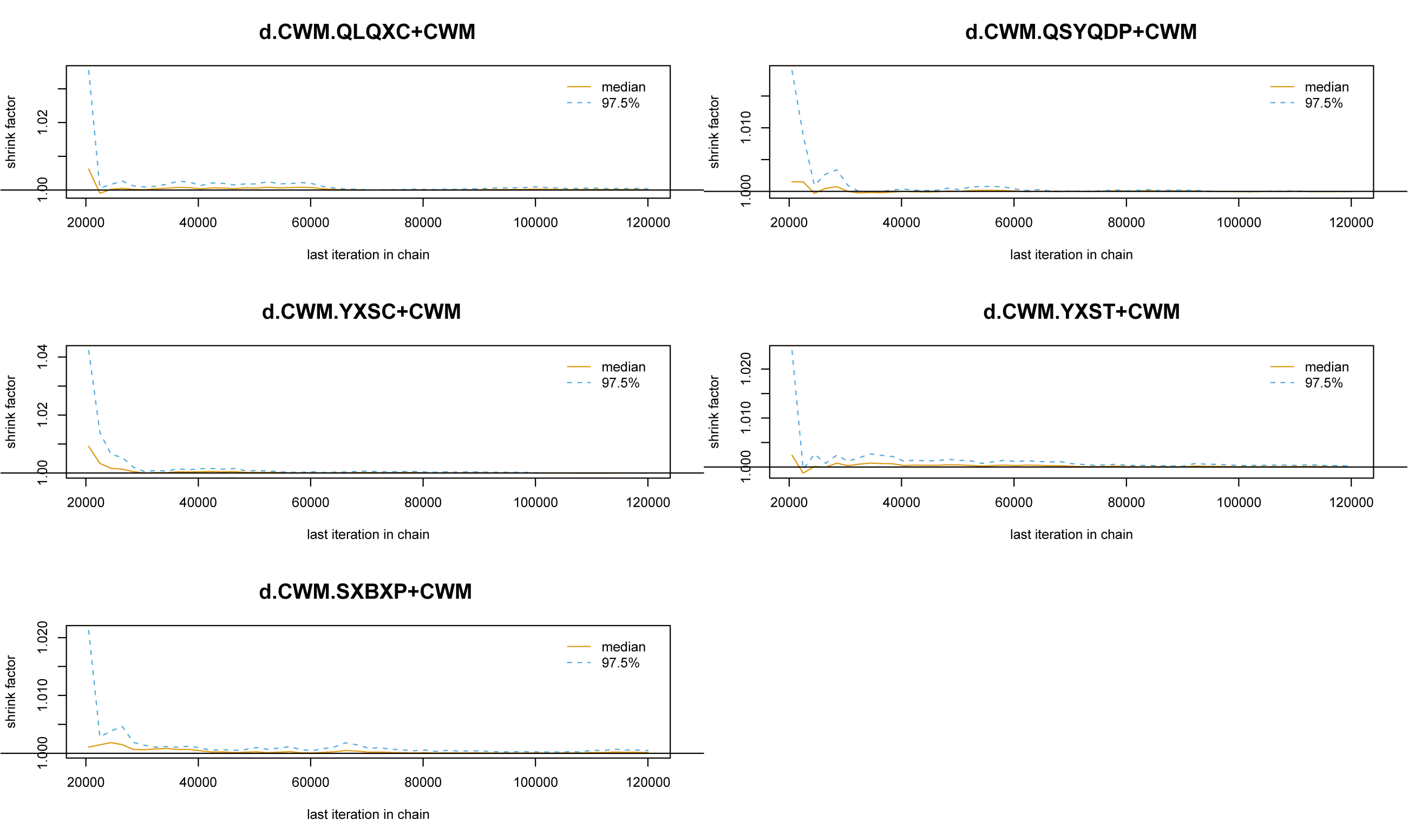


Brooks-Gelman-Rubin diagnosis plot for N-terminal pro-B type natriuretic peptide (NT-proBNP). CWM, conventional western medicine; QLQXC, Qili Qiangxin Capsule; QSYQDP, Qishen Yiqi dropping pill; YXSC, Yixinshu capsule; YXST Yangxinshi tablet; SXBXP, Shexiang Baoxin Pill.


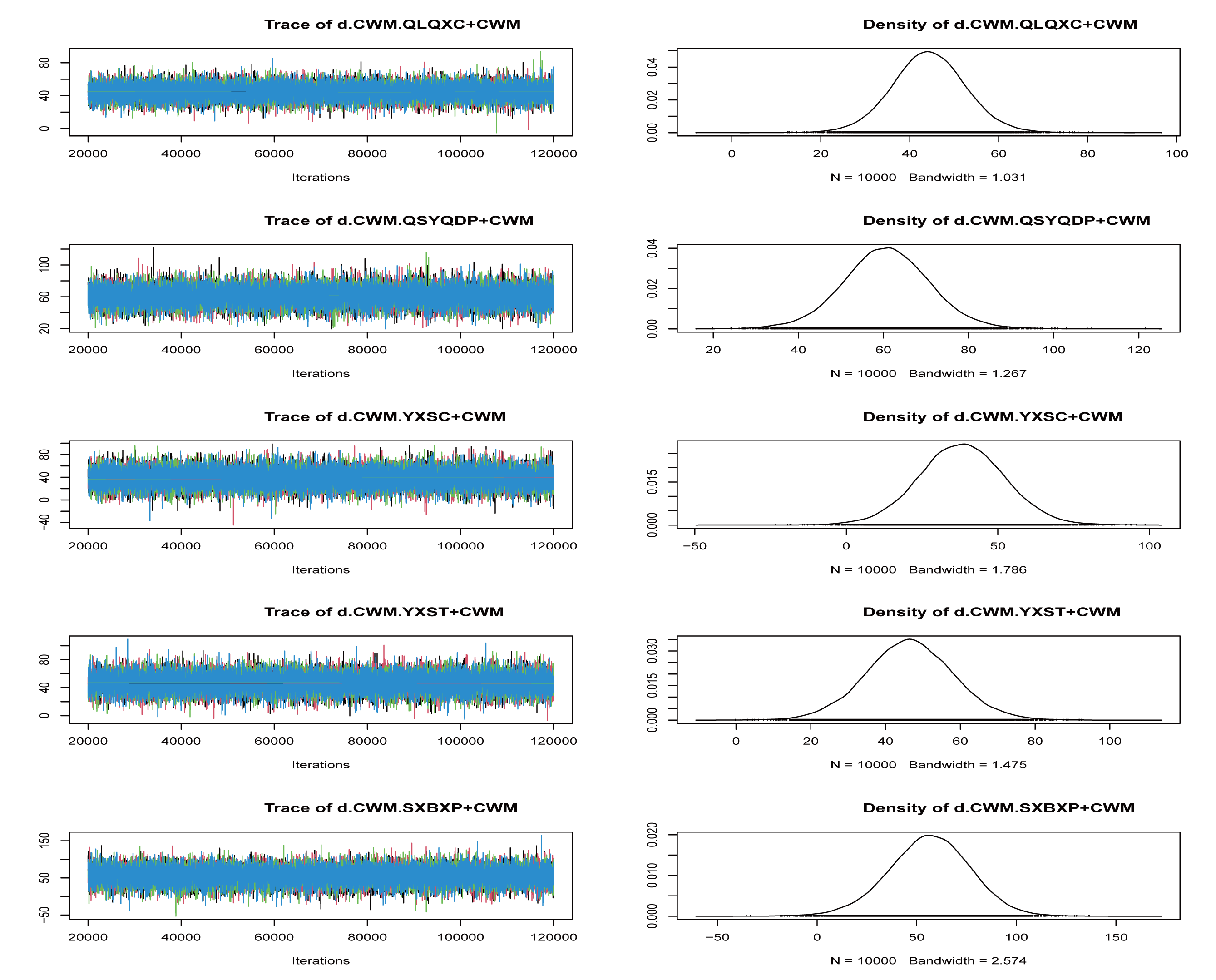


Trace plot and density plot for 6-minute walking test (6MWT). CWM, conventional western medicine; QLQXC, Qili Qiangxin Capsule; QSYQDP, Qishen Yiqi dropping pill; YXSC, Yixinshu capsule; YXST Yangxinshi tablet; SXBXP, Shexiang Baoxin Pill.


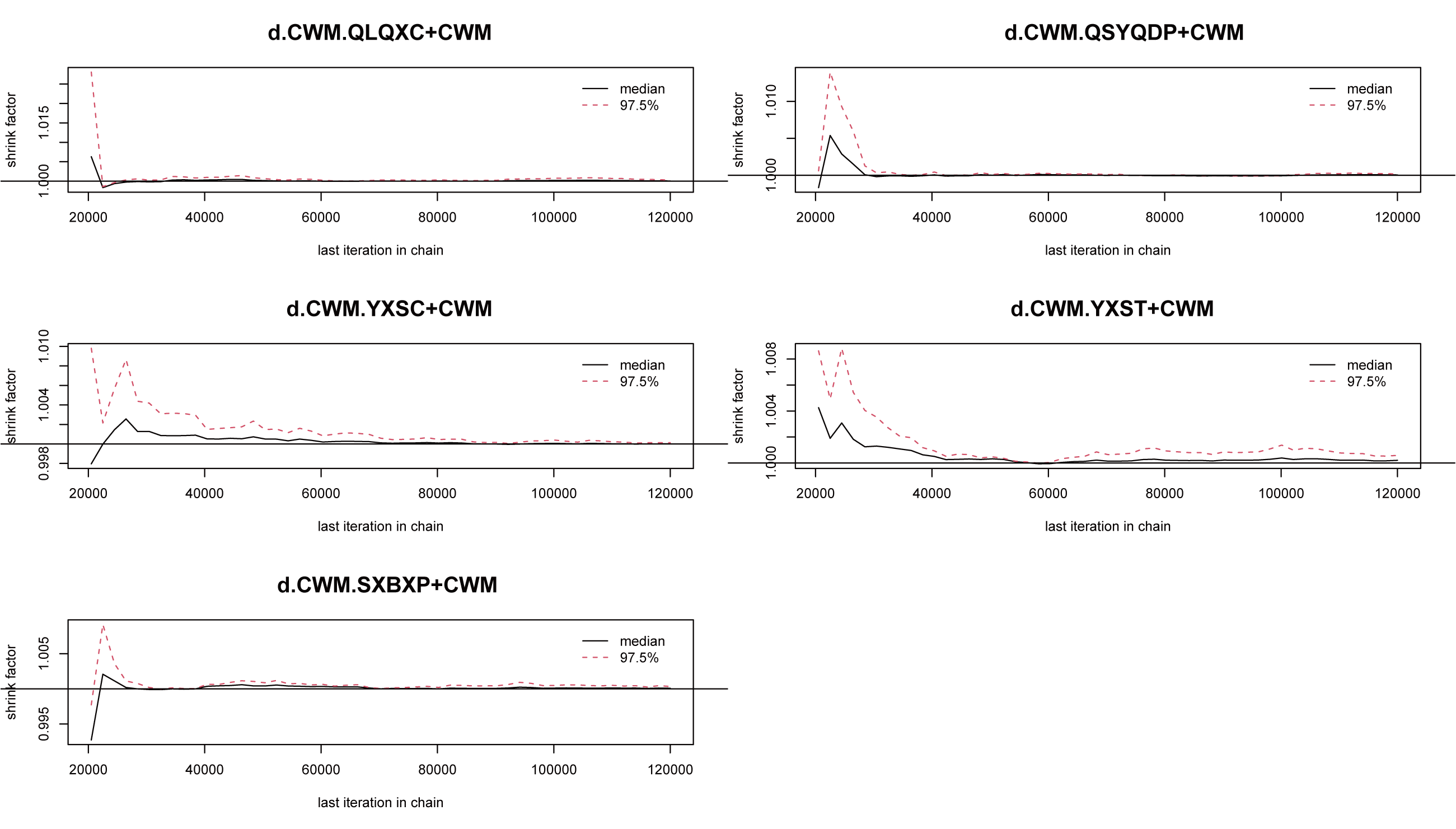


Brooks-Gelman-Rubin diagnosis plot for 6-minute walking test (6MWT). CWM, conventional western medicine; QLQXC, Qili Qiangxin Capsule; QSYQDP, Qishen Yiqi dropping pill; YXSC, Yixinshu capsule; YXST Yangxinshi tablet; SXBXP, Shexiang Baoxin Pill.
